# Effectiveness of Preanalytic Practices on Contamination and Diagnostic Accuracy of Urine Cultures: a Laboratory Medicine Best Practices Systematic Review and Meta-analysis

**DOI:** 10.1128/CMR.00030-15

**Published:** 2015-11-23

**Authors:** Mark T. LaRocco, Jacob Franek, Elizabeth K. Leibach, Alice S. Weissfeld, Colleen S. Kraft, Robert L. Sautter, Vickie Baselski, Debra Rodahl, Edward J. Peterson, Nancy E. Cornish

**Affiliations:** a M.T.L. Consulting, Erie, Pennsylvania, USA; b Kaiser Permanente, Los Angeles, California, USA; c Centers for Disease Control and Prevention, Atlanta, Georgia, USA; d Microbiology Specialists Incorporated, Houston, Texas, USA; e Emory University, Atlanta, Georgia, USA; f Carolinas Pathology Group, Carolinas Medical Center, Charlotte, North Carolina, USA; g University of Tennessee Health Science Center, Memphis, Tennessee, USA; h HealthEast Care System, St. Paul, Minnesota, USA; i Barnes Jewish Hospital, St. Louis, Missouri, USA

## Abstract

**Background.:**

Urinary tract infection (UTI) in the United States is the most common bacterial infection, and urine cultures often make up the largest portion of workload for a hospital-based microbiology laboratory. Appropriately managing the factors affecting the preanalytic phase of urine culture contributes significantly to the generation of meaningful culture results that ultimately affect patient diagnosis and management. Urine culture contamination can be reduced with proper techniques for urine collection, preservation, storage, and transport, the major factors affecting the preanalytic phase of urine culture.

**Objectives.:**

The purposes of this review were to identify and evaluate preanalytic practices associated with urine specimens and to assess their impact on the accuracy of urine culture microbiology. Specific practices included collection methods for men, women, and children; preservation of urine samples in boric acid solutions; and the effect of refrigeration on stored urine. Practice efficacy and effectiveness were measured by two parameters: reduction of urine culture contamination and increased accuracy of patient diagnosis. The CDC Laboratory Medicine Best Practices (LMBP) initiative's systematic review method for assessment of quality improvement (QI) practices was employed. Results were then translated into evidence-based practice guidelines.

**Search strategy.:**

A search of three electronic bibliographic databases (PubMed, SCOPUS, and CINAHL), as well as hand searching of bibliographies from relevant information sources, for English-language articles published between 1965 and 2014 was conducted.

**Selection criteria.:**

The search contained the following medical subject headings and key text words: urinary tract infections, UTI, urine/analysis, urine/microbiology, urinalysis, specimen handling, preservation, biological, preservation, boric acid, boric acid/borate, refrigeration, storage, time factors, transportation, transport time, time delay, time factor, timing, urine specimen collection, catheters, indwelling, urinary reservoirs, continent, urinary catheterization, intermittent urethral catheterization, clean voided, midstream, Foley, suprapubic, bacteriological techniques, and microbiological techniques.

**Main results.:**

Both boric acid and refrigeration adequately preserved urine specimens prior to their processing for up to 24 h. Urine held at room temperature for more than 4 h showed overgrowth of both clinically significant and contaminating microorganisms. The overall strength of this body of evidence, however, was rated as low. For urine specimens collected from women, there was no difference in rates of contamination for midstream urine specimens collected with or without cleansing. The overall strength of this evidence was rated as high. The levels of diagnostic accuracy of midstream urine collection with or without cleansing were similar, although the overall strength of this evidence was rated as low. For urine specimens collected from men, there was a reduction in contamination in favor of midstream clean-catch over first-void specimen collection. The strength of this evidence was rated as high. Only one study compared midstream collection with cleansing to midstream collection without cleansing. Results showed no difference in contamination between the two methods of collection. However, imprecision was due largely to the small event size. The diagnostic accuracy of midstream urine collection from men compared to straight catheterization or suprapubic aspiration was high. However, the overall strength of this body of evidence was rated as low. For urine specimens collected from children and infants, the evidence comparing contamination rates for midstream urine collection with cleansing, midstream collection without cleansing, sterile urine bag collection, and diaper collection pointed to larger reductions in the odds of contamination in favor of midstream collection with cleansing over the other methods of collection. This body of evidence was rated as high. The accuracy of diagnosis of urinary tract infection from midstream clean-catch urine specimens, sterile urine bag specimens, or diaper specimens compared to straight catheterization or suprapubic aspiration was varied.

**Authors' conclusions.:**

No recommendation for or against is made for delayed processing of urine stored at room temperature, refrigerated, or preserved in boric acid. This does not preclude the use of refrigeration or chemical preservatives in clinical practice. It does indicate, however, that more systematic studies evaluating the utility of these measures are needed. If noninvasive collection is being considered for women, midstream collection with cleansing is recommended, but no recommendation for or against is made for midstream collection without cleansing. If noninvasive collection is being considered for men, midstream collection with cleansing is recommended and collection of first-void urine is not recommended. No recommendation for or against is made for collection of midstream urine without cleansing. If noninvasive collection is being considered for children, midstream collection with cleansing is recommended and collection in sterile urine bags, from diapers, or midstream without cleansing is not recommended. Whether midstream collection with cleansing can be routinely used in place of catheterization or suprapubic aspiration is unclear. The data suggest that midstream collection with cleansing is accurate for the diagnosis of urinary tract infections in infants and children and has higher average accuracy than sterile urine bag collection (data for diaper collection were lacking); however, the overall strength of evidence was low, as multivariate modeling could not be performed, and thus no recommendation for or against can be made.

## INTRODUCTION

The most common infection occurring in the United States is urinary tract infection (UTI), accounting for nearly 7 million office visits, 1 million emergency room visits, and 100,000 hospitalizations per year (1, 2). Significantly more women than men are likely to experience UTIs, with 1 in 3 women having at least 1 episode of UTI necessitating treatment with antibiotics by the age of 24 (3). Nearly half of all women will experience at least one UTI during their lifetime (3–6). An increased risk of UTI occurs in certain population subgroups, including infants (7), pregnant women (8), the elderly (9), patients with spinal cord injuries and/or catheters (10), patients with diabetes (11) or multiple sclerosis (12), and patients with AIDS/human immunodeficiency virus (13, 14). The most common nosocomial infection is catheter-associated UTI, with over a million cases in hospital and nursing home patients every year (15). Increasing duration of catheterization increases the risk of infection (16). Urinary tract infections are the second-most-common infection in noninstitutionalized elderly populations and account for nearly 25% of all infections (9). The financial impact of UTIs is significant, with costs of up to $2 billion per year (17).

While many uncomplicated UTIs in outpatients are diagnosed clinically, the diagnosis of recurrent or complicated UTI is commonly achieved by testing urine specimens for the presence of microorganisms. As a result, urine cultures often make up the largest portion of the workloads of clinical microbiology laboratories (18). The appropriate management of components of the preanalytic phase of urine culture, namely, collection, preservation, and storage of urine specimens, has an important influence on the generation of meaningful culture results, which ultimately affects patient diagnosis and management (19).

### Quality Gap: Factors Associated with the Preanalytic Phase of Urine Culture

The major goal of proper specimen management is to ensure that specimen quality is maintained during collection and transport (20). Urine specimens can easily become contaminated with periurethral, epidermal, perianal, and vaginal flora. This contamination can be reduced with proper attention to techniques for urine collection, transport, preservation, and storage, the major components of the preanalytic phase of urine culture. A Q-Probe study conducted by the College of American Pathologists in 1998 (21) and again in 2008 (22) examined the frequency of urine culture contamination (defined as more than two isolates in quantities greater than 10,000 CFU/ml) and associated facility practices of urine collection and specimen management. Contamination rates of 41.7% (low-performance facilities), 15% (median performers), and 0.8% (high performers) correspond to the 10th, 50th, and 90th percentiles of facilities, respectively (22). Contamination rates had no correlation to collection site, use of collection kits, preservatives, or thermally insulated transport containers. However, contamination rates were substantially affected by postcollection processing, especially refrigeration of the specimen. Also, collection instructions given in the outpatient setting had a statistically significant impact on contamination rates in some cases. Based on the similarities of overall contamination rates between the two Q-Probe studies, the authors concluded that no significant progress in reducing urine culture contamination during the intervening years had been made (22). This may be a reflection of the inherent limitations of the Q-Probe methodology, which is based on one-time quality assessments dependent on the gathering of current data from large numbers of laboratories in order to establish provisional benchmarks for systematic quality improvement efforts. Many of these indicators are based primarily on self-reported surveys rather than on evidence-based scientific study designs and/or adequately specified, standardized, and consistently implemented data collection methods. Nonetheless, it is not cost-effective for laboratories to continue to waste valuable resources on the work-up of contaminated urine cultures (23). Furthermore, inappropriate reporting of contaminated urine cultures by the laboratory can result in patients receiving suboptimal or unnecessary therapy, producing poor patient outcomes and higher cost (18).

To address this important quality gap and its consequences, this research identified and evaluated practices associated with the collection, preservation, and storage of urine specimens for culture and their impact on the accuracy of urine culture microbiology. Rating criteria were used for evaluating these practices. Specific practices examined included collection methods for men, women, children, and infants; preservation of urine samples in boric acid solutions; and the effect of refrigeration on urine storage. The evidence supporting these practices for minimizing contaminated urine cultures and the impact on the accuracy of patient diagnosis were evaluated by applying the LMBP initiative's systematic review methods for quality improvement practices and by translating the results into evidence-based guidance (24). The methodology has recently been used to evaluate preanalytical practices for reducing blood culture contamination (25) and blood sample hemolysis (26).

## A-6 CYCLE FOR SYSTEMATIC REVIEW

The CDC's LMBP “A-6 Cycle” systematic review methods for evaluating quality improvement practices was used for conducting this review. The methodology, reported in detail elsewhere (24), is derived from previously validated methods. It is designed to assess the results of studies of practice effectiveness that lead to best-practice recommendations that are evidence based. Using this method, a review coordinator (author Mark T. LaRocco) and individuals trained to apply the LMBP methods (authors Alice S. Weissfeld and Elizabeth K. Leibach) conducted the systematic review with guidance from an expert panel. The expert panelists (authors Nancy E. Cornish, Colleen S. Kraft, Vickie Baselski, Robert L. Sautter, Edward J. Peterson, and Debra Rodahl) were chosen based on their breath of experience and perspective in clinical microbiology and laboratory management. A description of their scientific credentials and professional affiliations can be found in the author biography section. Lastly, the team was supported by a statistician with expertise in evidence review methodologies and meta-analysis (author Jacob Franek). The expert panel reviewed the results of the evidence review and drafted the evidence-based best-practice recommendations. The recommendations were then approved by the LMBP Workgroup, consisting of 13 invited members with broad expertise in laboratory medicine, clinical practice, health services research, and health policy, as well as one *ex officio* representative from the Centers for Medicare and Medicaid Services. A list of the members of the LMBP Workgroup is provided in Appendix 1.

### Review Question, Analytical Framework, and Search Strategy

**FIG 1:**
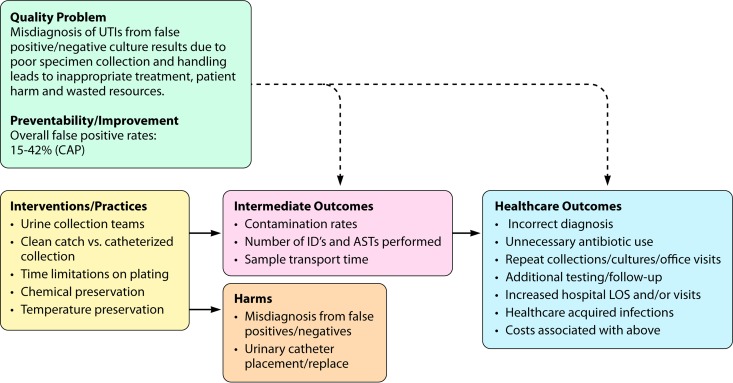
LMBP QI review question and analytic framework. Are there preanalytic practices related to the collection, storage, preservation, and transport of urine for microbiological culture that improve the diagnosis and management of patients with urinary tract infection? CAP, College of American Pathologists; ID's, identifications; ASTs, antimicrobial sensitivity tests; LOS, length of stay.

The review question addressed by this analytical review was as follows: “Are there preanalytic practices related to the collection, preservation, transport, and storage of urine for microbiological culture that improve the diagnosis and management of patients with urinary tract infection?” Components of the preanalytic phase of urine culture were studied in the context of an analytical framework for factors affecting specimen contamination and diagnostic accuracy, depicted in Fig. 1. The population, intervention, comparison, and outcome (PICO) elements are as follows.

- “Population” is any patients who have urine cultures collected.
- “Intervention” is clinical practice.
- “Comparison” is made of
  - immediate versus delayed processing of urine held at room temperature,
  - immediate versus delayed processing of refrigerated urine or urine preserved in boric acid,
  - midstream clean-catch collection of urine without cleansing versus with cleansing (men and women),
  - midstream clean-catch collection of urine without cleansing versus with cleansing versus collection with a sterile urine bag versus diaper collection for infants and children.
- “Outcomes” are the results of determining the contamination rate and the diagnostic accuracy of urine culture.

Specific practices involving the preanalytic phase of urine culture covered in this evidence-based review were addressed by asking the following eight clinical questions.

1. What is the difference in colony counts when comparing immediate versus delayed processing of fresh urine stored at room temperature after collection?
2. What is the difference in colony counts when comparing immediate versus delayed processing of urine kept refrigerated or preserved in boric acid?
3. What is the difference in contamination rates between midstream urine collected with cleansing versus without cleansing in women being tested for a UTI?
4. What is the diagnostic accuracy of midstream urine collected with or without cleansing compared to bladder catheterization for the diagnosis of UTI in women?
5. What is the difference in contamination rates between midstream urine collection, with or without cleansing, and first-void collection in men?
6. What is the diagnostic accuracy of midstream urine collected, with or without cleansing, compared to that of bladder catheterization or suprapubic aspiration for the diagnosis of UTI in men?
7. What are the differences in contamination rates between midstream collection with cleansing, midstream collection without cleansing, and sterile urine bag or diaper collection in children?
8. What is the diagnostic accuracy of midstream clean-catch, sterile urine bag, or diaper collection compared with that of suprapubic aspiration or catheterization for the diagnosis of UTI in children?

The search for studies of practice effectiveness was conducted to identify those with measurable outcomes collected to the rigor of review requirements. With input from the expert panel and assistance of a research librarian at the Jesse Jones Library at the Texas Medical Center in Houston, TX, a literature search strategy and set of terms were developed. A search of three electronic bibliographic databases (PubMed, SCOPUS, and CINAHL) for English-language articles published between 1965 and 2014 was conducted. In addition, hand searching of bibliographies from relevant information sources was performed. All search results were catalogued and maintained using a Web-based, commercial reference software package (RefWorks; ProQuest LLC, Ann Arbor, MI). Finally, solicitation of unpublished quality improvement studies was attempted by posting requests for data on both the Laboratory Medicine Best Practices website (https://wwwn.cdc.gov/futurelabmedicine/) and two listservs supported by the American Society for Microbiology: clinmicronet (http://www.asm.org/index.php/online-community-groups/listservs) and DivCNet (http://www.asm.org/division/c/divcnet.htm).

The search contained the following medical subject headings (MESH) and key text words: “urinary tract infections” (MESH) OR UTI (text word) OR urinary tract infect* (text word); “urine/analysis” (major) OR “urine/microbiology” (major) OR “urinalysis” (MESH); “specimen handling” (major); “preservation, biological” (MESH) OR preservation, biological (text word) OR “boric acids” (MESH) OR boric acid (text word) OR boric acid/borate (text word) OR boric acids (text word) OR “refrigeration” (MESH) OR refrigeration (text word) OR preserv* (text word); storage (text word); “time factors” (MESH) OR “transportation” (MESH) OR transport time (text word) OR delay (text word) OR time delay (text word) OR time factor (text word) OR timing (text word); “urine specimen collection” (MESH) OR urine specimen collection (text word) OR “catheters, indwelling” (MESH) OR catheters, indwelling (text word) OR “urinary reservoirs, continent” (MESH) OR urinary reservoirs, continent (text word) OR “urinary catheterization” (MESH) OR urinary catheterization (text word) OR “intermittent urethral catheterization” (MESH) OR intermittent urethral catheterization (text word) OR clean voided (text word) OR midstream (text word) OR midstream (text word) OR midstream (text word) OR foley (text word) OR suprapubic (text word); and “bacteriological techniques” (MESH) OR bacteriological technique (text word) OR bacteriological techniques (text word) OR “microbiological techniques” (MESH) OR microbiological technique (text word) OR microbiological techniques (text word).

Titles and abstracts were initially screened by the review coordinator, with assistance from the expert panel when necessary, to select studies for a full review. A study was included if it was considered likely to provide valid and useful information and met the PICO criteria previously discussed. Specifically, these inclusion criteria required that a study (i) address a defined population/definable group of patients, (ii) evaluate a specific intervention/practice included in this review, (iii) describe at least one finding for a relevant outcome measure (percent contamination, diagnostic accuracy) reproducible in comparable settings, and (iv) present results in a format which was useful for statistical analysis. Studies failing to meet the inclusion criteria (not considered to report a relevant practice, did not include a practice of interest, or did not present an outcome measure of interest) were excluded from further review.

Studies that cleared this initial screening were then abstracted and evaluated by the expert panel. For eligible studies, information on study characteristics, interventions, outcome measures, and findings of the study was extracted using a standardized form and assigned a quality rating derived from points awarded for meeting quality criteria. Individual quality ratings were based on four dimensions: study quality, practice effectiveness, defined outcome measure(s), and findings/results. The objective for rating individual study quality was to judge whether sufficient evidence of practice effectiveness was available to support inclusion in an overall body of evidence for evaluation of a best-practice recommendation (that is, a practice likely to be effective in improving one or more outcomes of interest in comparison to other commonly used practices).

The four study quality dimensions were rated separately, with a rating score assigned up to the maximum for a given dimension. The rating scores for all four dimensions were added to reach a single summary score reflecting overall study quality. A total of 10 points were available for each study. Reviewers assigned one of three quality ratings to each study: good (8 to 10 points), fair (5 to 7 points), or poor (4 points or less). Each study was reviewed and rated by two expert panel members to minimize subjectivity and bias. Any study ranked as poor by one reviewer but good by the second reviewer was assigned to a third expert panel member for resolution. More detail on the rating process of individual studies can be found elsewhere (24–26). Studies that did not meet a study quality rating of fair or good were excluded from further consideration. Data from published studies that passed a full review were transformed to a standardized, common metric according to LMBP methods (24). Summary data and quality scores for each publication included in this evidence-based review can be found in Appendix 3 below.

The study quality ratings and results from the individual studies for each clinical question were aggregated into bodies of evidence. The consistency of effects and patterns of effects across studies and the rating of overall strength of the body of evidence (high, moderate, low, suggestive, and insufficient) were based on both qualitative and quantitative analyses. Estimates of effect and the strength of the body of evidence were then used to translate results into one of three evidence-based recommendations (recommend, no recommendation for or against, recommend against). The ratings criteria are described in greater detail elsewhere (24).

While recommendations are based on the entire body of evidence, meta-analyses to generate summary estimates of effect were undertaken for outcomes that provided sufficient data for measurements of diagnostic accuracy and contamination, i.e., proportions of specimens containing periurethral, perianal, epidermal, or vaginal flora. For the outcome of contamination proportion, summary odds ratios were calculated using Mantel-Haenszel methods in a random-effects model performed using Review Manager (RevMan) software version 5.0 (2008; The Nordic Cochrane Centre, The Cochrane Collaboration, Copenhagen, DK). A contamination event was defined according to how individual studies defined contamination because definitions varied between studies. Wherever possible, contamination proportions were determined for the entire test population rather than a subset population (such as only among those individuals that tested negative for urinary tract infection). The I^2^ statistic, which describes the percentage of variability in effects estimates due to statistical heterogeneity rather than sampling error, was used to assess between-study heterogeneity. For the outcomes of diagnostic accuracy, it was planned that point estimates of sensitivity and specificity would be summarized using the bivariate model when similar cutoff points were used; however, all models failed to converge due to a too-small number of study or sample sizes. Similarly, hierarchical summary receiver operator characteristic curves (HSROC) could not be generated because these models too failed to converge. Solutions for failure of convergence, including removing individual studies, were explored but did not improve convergence. Meta-analysis of diagnostic accuracy outcomes and curve fitting were not pursued further given the limitations of univariate methods. All work on summarizing diagnostic accuracy outcomes was performed using SAS software version 9.2 (2008; SAS Institute Inc., Cary, NC, USA) and the MetaDAS macro, version 1.3 (27). Significant growth (i.e., a positive sample) was defined according to how each individual study defined significant growth because cutoff points tended to vary among studies. All other growth, including contamination and no growth, were considered nonsignificant growth (i.e., a negative sample), as this most closely reflects actual clinical practice. Two-by-two tables were used to determine sensitivity and specificity, and exact 95% confidence intervals were calculated.

### Search Results

**FIG 2:**
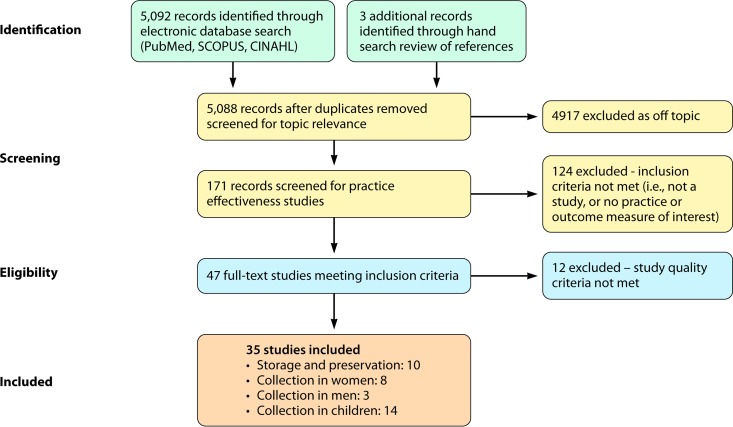
Systematic review flow diagram.

Search results produced 5,092 unique documents that were initially screened for eligibility to contribute to evidence of effectiveness for practices defined by the eight clinical questions posed (storage and preservation of urine, collection of urine from women, collection of urine from men, and collection of urine from infants and children). There was no response to requests for unpublished data. The reduction of studies through the screening process is detailed in Fig. 2. Initial screening for topic relevance eliminated 4,917 studies. From the remaining 171 studies, 124 were eliminated for not meeting the inclusion criteria (i.e., having elements potentially relevant to at least one topic area review question, reporting practices that are in use and available for adoption, reporting practices reproducible in other comparable settings, and addressing a defined population/definable group of patients). Forty-seven studies met the criteria for inclusion and were subjected to full abstraction and quality scoring. After an additional 12 studies were excluded because of insufficient quality scores, the remaining 35 were included in the statistical analysis: 10 studies on storage and preservation, 8 studies on collection from women, 3 studies on collection from men, and 14 studies on collection from infants and children.

## STORAGE AND PRESERVATION OF URINE

Summary information on the 10 published studies comprising the body of evidence for the clinical questions on the storage and preservation of urine is presented in Tables 1 and 2. The publication dates for these studies range from 1969 (28) to 1999 (29). All studies were given a “fair” quality rating. Three studies examined the effect of prolonged storage of clean-catch urine at room temperature by culturing samples of urine immediately upon receipt and then again after 2 h and 4 h (30), after 4 h, 24 h, and 48 h (31), or after 24 h and 72 h (28) of storage at room temperature. Nine studies tested the effect of preserving urine in boric acid for 24 h on colony counts and compared the results with the results of immediate culture. Several different boric acid formulations were used, including boric acid alone (29, 31, 32), glycerol-boric acid-sodium formate (32–37), and sorbitol-boric acid-sodium formate (32, 37). The length of delay of culture while samples were preserved in boric acid was assessed at various time points across studies, but 24 h was chosen for analysis as it was the most common endpoint. Three studies examined the effect of 24-h refrigeration of urine samples on changes in colony counts from those of immediate culture (33, 35, 37). The majority of studies used clean-catch midstream urine samples, although collection methods were undefined in five studies (30, 32, 34, 35, 37). Growth was defined as either “significant” or “nonsignificant.” The definitions of significant growth varied among studies, but in general, a threshold of >10^5^ CFU/ml of one or two species of bacteria was used.

**TABLE 1:** Body-of-evidence table for clinical question 1, namely, “what is the difference in colony counts when comparing immediate and delayed (≥4 h) processing of fresh urine stored at room temperature after collection?”^*a*^

Study (reference), quality rating	Samples	Setting	Time period	Results
Hindman et al. (30), fair	100 random samples of urine were cultured within 2 h of collection and then again after 2 h and 4 h of storage at room temp.	Clinical Microbiology Laboratory, Hartford Hospital, Hartford, CT	Not given	SG was defined as any growth of >10^5^ CFU/ml. All other growth was considered NSG. Upon receipt, there were 47 SG and 53 NSG specimens. After 4 h, there were 51 SG and 49 NSG specimens.
Lum and Meers (31), fair	175 clean-catch urine samples were divided, and one portion was treated with boric acid at a concn of 20 g/liter and the other held in a sterile tube. All samples were cultured upon receipt in the laboratory and again after 4 h, 24 h, and 48 h of storage at room temp.	Microbiology Department, University of Singapore, Kent Ridge, Singapore	6 mo	SG was defined as ≥10^5^ CFU/ml of 1 or 2 species. All other growth was considered NSG. Upon receipt, there were 38 SG and 137 NSG specimens. At 4 h, there were 42 SG and 133 NSG specimens. At 24 h, there were 90 SG and 82 NSG specimens. At 48 h, there were 109 SG and 66 NSG specimens.
Porter and Brodie (28), fair	130 midstream urine specimens that had been collected in sterile tubes and kept at room temp or preserved with 0.5 g of boric acid were mailed to a laboratory and cultured immediately upon receipt (avg delay of 24 h before receipt) and again at 72 h after receipt.	Laboratory, City Hospital, Aberdeen, Scotland	Not given	SG was defined as any growth of >10^5^ CFU/ml. All other growth was considered NSG. Upon receipt, there were 40 SG and 90 NSG specimens. After 72 h, there were 93 SG and 37 NSG specimens.

a SG, significant growth; NSG, nonsignificant growth.

**TABLE 2:**
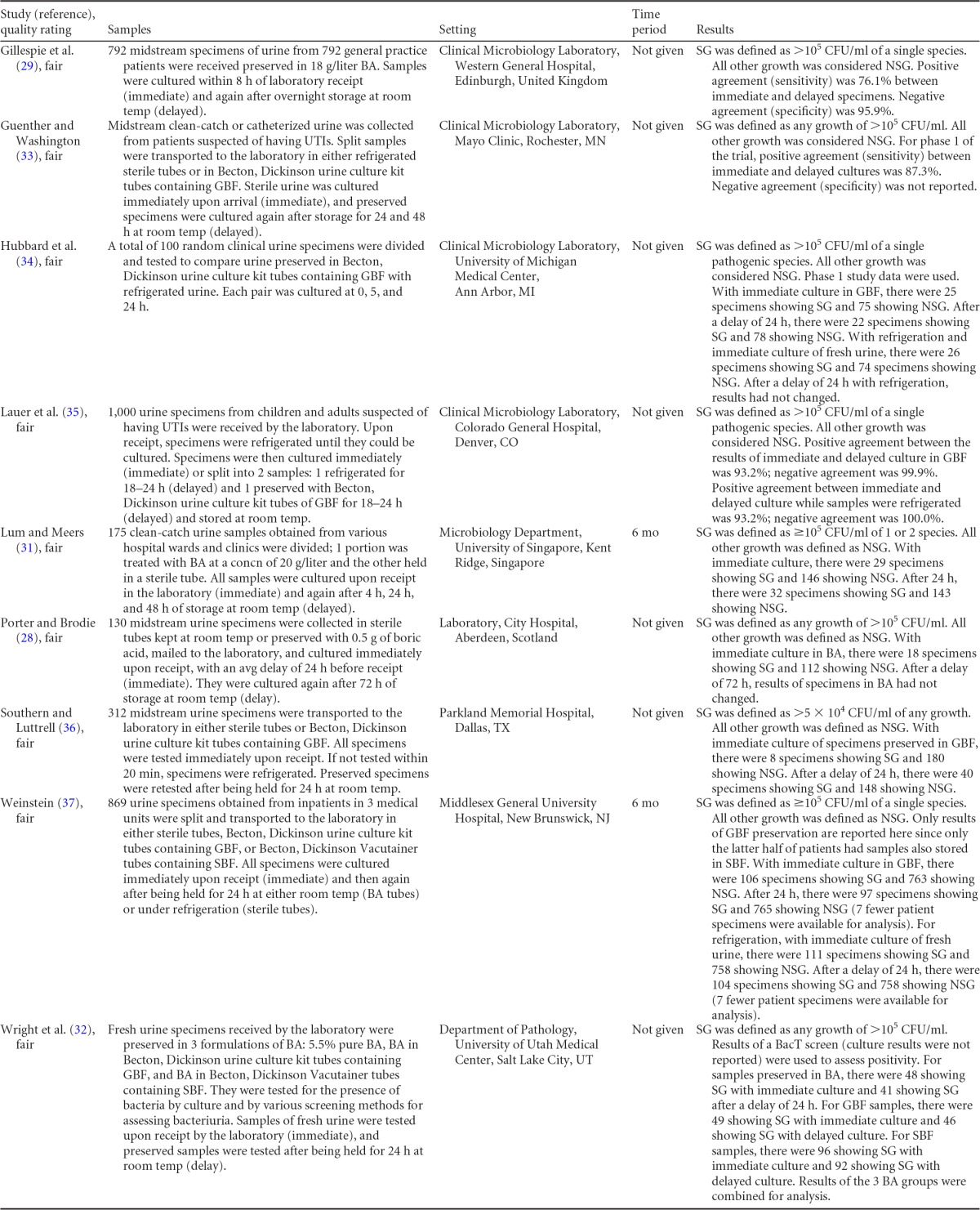
Body-of-evidence table for clinical question 2, namely, “what is the difference in colony counts when comparing immediate and delayed (≥24 h) processing of urine kept refrigerated or preserved in boric acid?”^*a*^

a BA, boric acid; GBF, glycerol-boric acid-sodium formate; SBF, sorbitol-boric acid-sodium formate; SG, significant growth; NSG, nonsignificant growth.

### Body-of-Evidence Qualitative Analysis

The difference in colony counts when immediate and delayed processing of urine specimens stored at room temperature were compared is shown in Table 3. Data from three observational studies (28, 30, 31) found a moderate increase (approximately 10%) in colony counts after 4 h of storage at room temperature and a large increase (>135%) in colony counts after storage for 24 h or more. The effect of delayed culture on urine specimens kept refrigerated or preserved in solutions of boric acid is shown in Tables 4, 5, and 6. Data from three observational studies (29, 33, 35) found 73 to 93% positive agreement (sensitivity) and 96 to 100% negative agreement (specificity) between the results of immediate culture and after a 24-h delay with specimens preserved in boric acid. Data from one study (35) found 93% positive agreement and 100% negative agreement between specimens cultured immediately upon receipt versus after a 24-h delay with refrigeration (Table 4). Colony counts in urine samples either refrigerated or chemically preserved showed similar results. Five studies (31, 32, 34, 37, 38) showed that urine samples preserved in boric acid solutions for 24 h (Table 5) or refrigerated for 24 h (Table 6) had only minor changes in the numbers of cultures with either significant or nonsignificant growth.

**TABLE 3:** Difference in colony counts when results of immediate and delayed plating of fresh urine stored at room temperature were compared^*a*^

Study (reference)	No. of organisms at 0 h (CFU/ml)	Increase in significant growth (%) at:			
		4 h	24 h	48 h	72 h
Lum and Meers (31)	38	11	137	187	ND
Hindman et al. (30)	47	9	ND	ND	ND
Porter and Brodie (28)	40	ND	ND	ND	233

a The quality rating of each study was fair. ND, not determined.

**TABLE 4:** Results of immediate versus delayed culture of urine preserved in boric acid or refrigerated^*a*^

Study (reference)	Preservative	Time zero storage conditions	Storage conditions for delayed culture	Positivity threshold (CFU/ml)	% sensitivity (95% CI)	% specificity (95% CI)
Lauer et al. (35)	GBF	Refrigeration	18–24 h in GBF	>10^5^	93 (86–97)	100 (99–100)
Gillespie et al. (29)	BA	<8 h in BA	Overnight in BA	>10^5^	76 (68–82)	96 (94–97)
Guenther and Washington (33)	GBF	Refrigeration	24 h in GBF	≥10^5^	87 (78–93)	ND
Lauer et al. (35)		Refrigeration	18–24 h of refrigeration	>10^5^	93 (86–97)	100 (100–100)

a The quality rating of each study was fair. GBF, glycerol-boric acid-sodium formate; BA, boric acid; 95% CI, 95% confidence interval; ND, not determined.

**TABLE 5:** Effect of delayed plating of urine specimens preserved in boric acid solutions^*a*^

Study (reference)^*b*^	Preservative(s)	Preservative used for immediate culture	No. of h that culture was delayed (preservative[s])	Threshold (no. of CFU/ml)	NSG present		SG present	
					No. of specimens subjected to immediate culture	% change from no. after delay	No. of specimens subjected to immediate culture	% change from no. after delay
Southern and Luttrell (36)	GBF	GBF	24 (GBF)	>5 × 10^4^	180	−17.8	8	+500.0
Lum and Meers (31)	BA	GBF	24 (GBF)	>10^5^	146	−2.1	29	+10.3
Wright et al. (32)	BA, GBF, SBF	None (fresh specimens were used)	24 (BA, GBF, SBF)	>10^5^			193	−7.3
Weinstein (37)	GBF, SBF	GBF	24 (GBF)	≥10^5^	763	+1.2^*b*^	106	−8.5^*b*^
Hubbard et al. (34)	GBF	GBF	24 (GBF)	>10^5^	75	+4.0	25	−12.0
Porter and Brodie (28)	BA	BA	72 (BA)	>10^5^	112	0	18	0

a GBF, glycerol-boric acid-formate; BA, boric acid; SBF, sorbitol-boric acid-formate; NSG, nonsignificant growth; SG, significant growth. All studies were given a quality rating of fair.

b There were 7 fewer patient samples available for analysis with delayed culture (862 patient pairs versus 869); the percent increase was calculated assuming 869 pairs.

**TABLE 6:** Effect of delayed plating of urine specimens kept refrigerated^*a*^

Study (reference)	Threshold (no. of CFU/ml)	NSG present		SG present	
		No. of specimens subjected to immediate culture	% change from no. after delay	No. of specimens subjected to immediate culture	% change from no. after delay
Weinstein (37)	≥10^5^	758	+0.9^*b*^	111	−6.3^*b*^
Hubbard et al. (34)	>10^5^	74	0	26	0

a Both studies were given a quality rating of fair. NSG, nonsignificant growth; SG, significant growth. Both studies immediately cultured fresh specimens and specimens that had been kept under refrigeration for 24 h.

b There were 7 fewer patient samples available for analysis with delayed culture (862 patient pairs versus 869); percentages of increase were calculated assuming 869 pairs.

These data suggest that both boric acid and refrigeration adequately preserve urine specimens prior to their processing for up to 24 h. Furthermore, the results suggest that urine held at room temperature for more than 4 h should not be processed due to overgrowth of both clinically significant and contaminating microorganisms. Based on statistical analysis of the data, however, the overall strength of this body of evidence was rated as low.

## COLLECTION OF URINE FROM WOMEN

Summary information on the eight published studies comprising the body of evidence for the clinical questions on contamination rates and the diagnostic accuracy of midstream urine collection from adult females is presented in Tables 7 and 8. Three studies (39–41) were given a quality rating of “good,” and five studies (38, 42–45) were rated as “fair.” Five studies (38–40, 42, 43) examined the impact of perineal cleansing on contamination and are summarized in Table 7. Patient settings included a clinic for adolescents (38), a general practice (38), an antenatal ambulatory-care clinic (39, 43), and a health center for teenagers (40). Definitions of contamination varied among studies and included any growth of normal vaginal flora and/or small quantities (<2,000 CFU/ml) of pathogenic bacteria (38), the presence of epithelial cells (42), mixed growth in quantities of >10^5^ CFU/ml (39) or at any quantity (43), and growth of any nonpathogen or pathogen in quantities of <10^4^ CFU/ml (43) or <10^5^ CFU/ml (40).

**TABLE 7:**
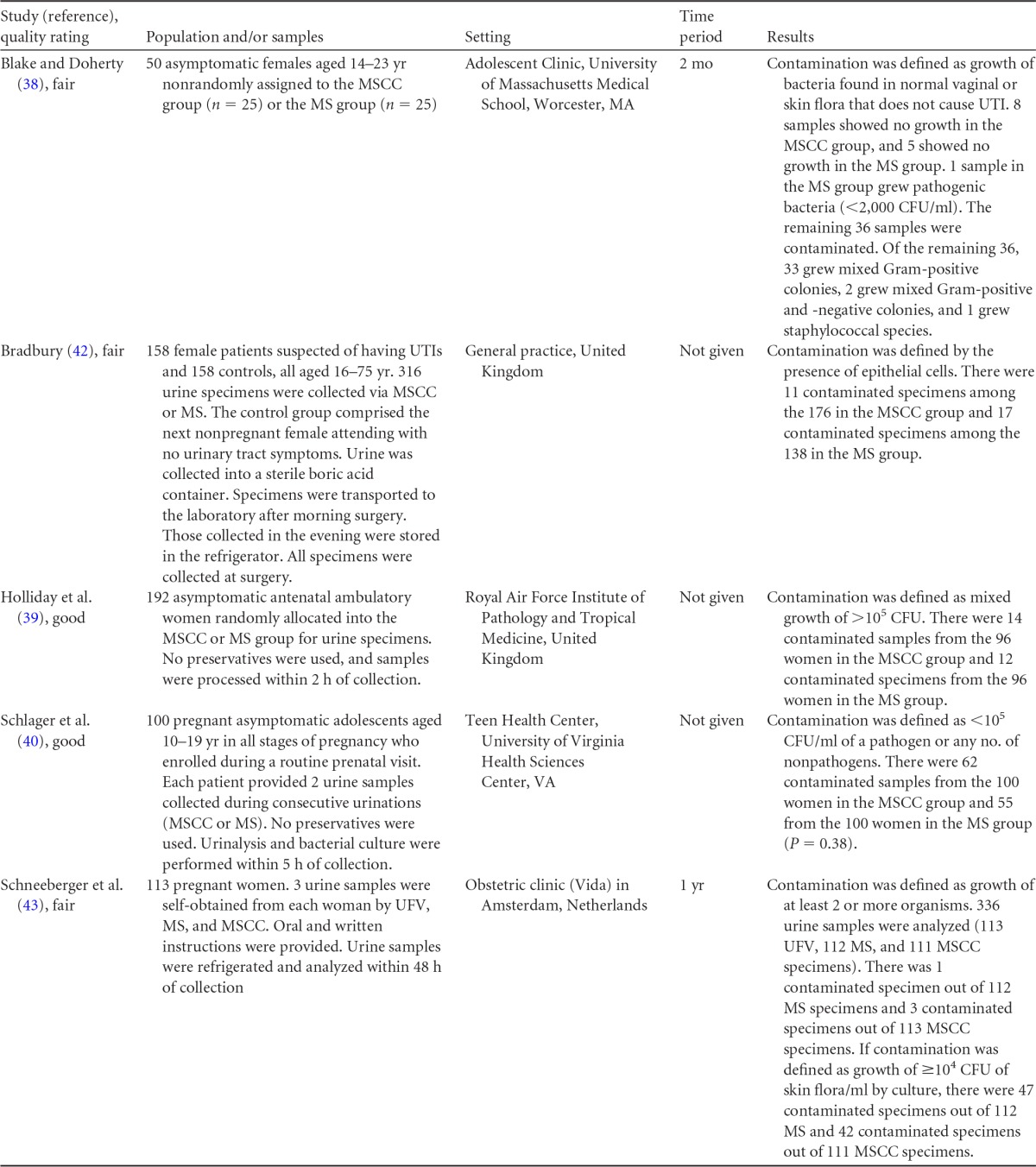
Body-of-evidence table for clinical question 3, namely, “what is the difference in percentages of contamination between midstream urine collection with cleansing versus without cleansing in women being tested for a UTI?”^*a*^

a MSCC, midstream collection with perineal cleansing; MS, midstream collection; UFV, first-void urine collection (morning).

**TABLE 8:**
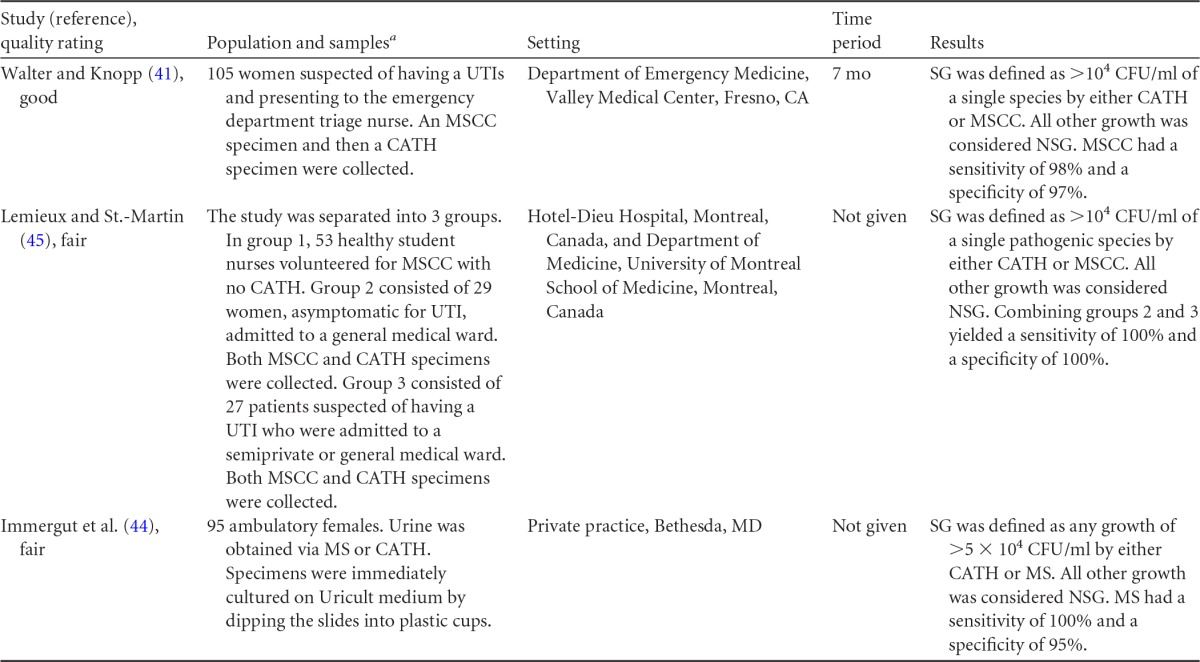
Body-of-evidence table for clinical question 4, namely, “what is the accuracy of midstream urine collection, with or without cleansing, compared to catheterization for the diagnosis of UTI in women?”^*a*^

a MSCC, midstream collection with perineal cleansing; MS, midstream collection; CATH, catheterization.

Three studies (41, 44, 45) examined the diagnostic accuracy of midstream urine collection with or without cleansing, with straight urinary catheterization as the reference standard (Table 8). Patient populations in these studies included women presenting to an emergency department (41) or ambulatory clinic (44) or admitted to a general medical ward (45). In two studies (43, 44), each patient had urine collected by midstream collection with cleansing, followed by a second collection by urinary catheterization. In the third study (46), no cleansing was performed prior to midstream collection.

### Body-of-Evidence Qualitative Analysis

**FIG 3:**
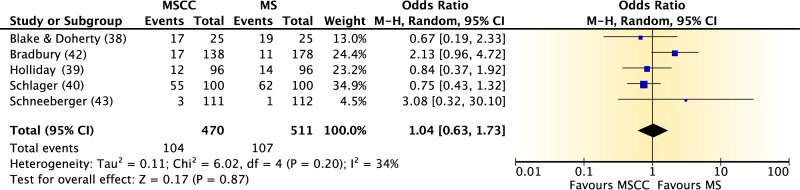
Difference in contamination levels between midstream urine collected with cleansing (MSCC) versus without cleansing (MS) in women being tested for urinary tract infection. M-H, Mantel-Haenszel statistic; 95% CI, 95% confidence interval.

The evidence examining the impact of perineal cleansing on contamination of midstream urine specimens collected from females is depicted in Fig. 3. Data from four observational studies (38, 40, 42, 43) and one randomized control trial (39) found no difference in the odds of contamination between midstream urine specimens collected with or without cleansing. The overall strength of this evidence was rated as high. The diagnostic accuracy of midstream urine collection with or without cleansing is shown in Table 9. Using catheterization as the reference standard, midstream collection had a sensitivity of 98 to 100% and a specificity of 95 to 100%. However, the overall strength of this body of evidence was rated as low.

**TABLE 9:** Accuracy of midstream clean-catch or midstream urine collection compared to catheterization for the diagnosis of UTI in women^*a*^

Study (reference)	Quality rating	Subpopulation	Index test	Positive threshold for reference standard (CFU/ml)	Positive threshold for index test (CFU/ml)	% sensitivity (95% CI)	% specificity (95% CI)
Walter and Knopp (41)	Good	ND	MSCC	>10^4^	>10^4^	98 (88–100)	97 (89–99)
Lemieux and St.-Martin (45)	Fair	Combined	MSCC	>10^4^	>10^4^	100 (87–100)	100 (89–100)
		Asymptomatic	MSCC	>10^4^	>10^4^	ND	100 (88–100)
		Symptomatic	MSCC	>10^4^	>10^4^	100 (44–100)	95 (88–98)
Immergut et al. (44)	Fair	ND	MS	>5 × 10^4^	>5 × 10^4^	100 (44–100)	95 (88–98)

a MSCC, midstream clean-catch collection; MS, midstream urine collection; ND, not determined. The reference standard for all tests was catheterization.

## COLLECTION OF URINE FROM MEN

Summary information on the three published studies comprising the body of evidence for the clinical questions on contamination and diagnostic accuracy of midstream urine collection from adult males is presented in Tables 10 and 11. One study (46) was given a quality rating of “good,” and two studies (47, 48) were rated as “fair.” Two studies (46, 47) examined contamination in midstream clean-catch specimens compared to that in first-void collection specimens (Table 10). Patients in both studies were either ambulatory or hospitalized men with symptoms of urinary tract infection being seen at a VA Medical Center. In the first study (46), men had a first-void and/or midstream urine sample collected, but only half of the patients were asked to wash their glans penis prior to collection. In the second study (47), urine specimens from men were obtained by midstream clean-catch collection, first-void collection, straight catheterization, and suprapubic bladder aspiration, with 7 men being sampled more than once. Contamination was defined as either the growth of >10^3^ CFU/ml of two or more colony types (46) or any growth of three or more microbial species (47). For the meta-analysis, only those samples obtained via midstream clean-catch collection and first-void collection without cleansing were compared.

**TABLE 10:**
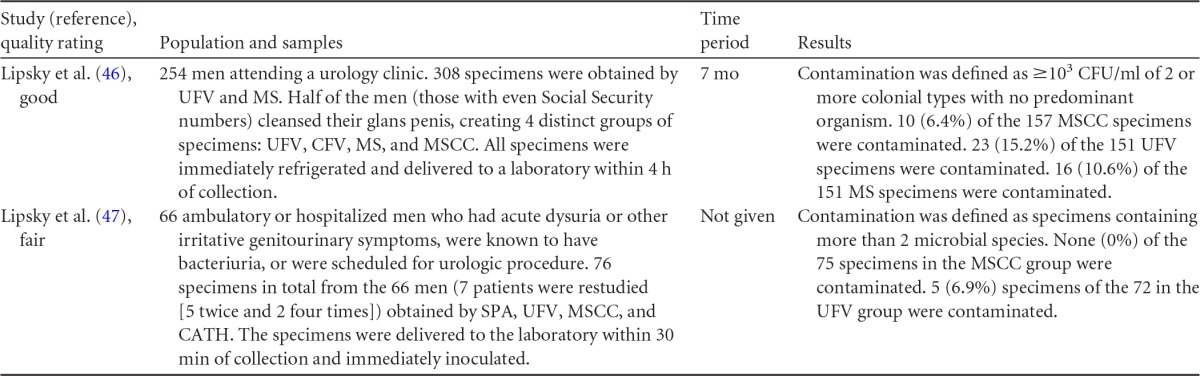
Body-of-evidence table for clinical question 5, namely, “what is the difference in contamination between midstream urine collection, with or without cleansing, from first-void collection from men?”^*a*^

a MS, unclean midstream urine collection; MSCC, midstream clean-catch collection; UFV, first-void urine collection without cleansing; CFV, first-void collection with cleansing; SPA, suprapubic aspiration; CATH, urethral catheterization. The setting for these studies was the VA Medical Center, Seattle, WA.

**TABLE 11:** Body-of-evidence table for clinical question 6, namely, “what is the accuracy of midstream urine collection compared to straight catheterization or suprapubic aspiration for the diagnosis of UTI in men?”^*a*^

Study (reference), quality rating	Population and samples	Setting(s)	Time period	Results
Lipsky et al. (47), fair	66 ambulatory or hospitalized men who had acute dysuria or other irritative genitourinary symptoms, were known to have bacteriuria, or were scheduled for a urologic procedure. 76 specimens in total were obtained from the 66 men (7 patients were restudied [5 twice and 2 four times]) obtained by SPA, UFV, MSCC, and CATH. Specimens were delivered to the laboratory within 30 min of collection and immediately inoculated.	VA Medical Center, Seattle, WA	Not given	SG was defined as ≥10^4^ CFU/ml of a single or predominant species (≥90% of the plate's growth) for MSCC and ≥10^3^ for SPA/CATH. All other growth was considered NSG. MSCC had a sensitivity of 82.4% and a specificity of 100.0%.
Deresinski and Perkash (48), fair	53 male spinal cord injury patients who were free of indwelling catheters. 71 samples of urine were obtained, 1 by MSCC and 1 by SPA. Note that many of the MSCC specimens were collected on first void. Urine specimens were processed for culture immediately.	Spinal Cord Injury Service, VA, and Stanford University Medical Centers, Palo Alto, CA	Not given	SG was defined as any growth of >10^4^ CFU/ml for MSCC and SPA. All other growth was considered NSG. MSCC had a sensitivity of 100% and a specificity of 100%.

a MSCC, midstream clean-catch collection; UFV, first-void urine collection without cleansing; SPA, suprapubic aspiration; CATH, urethral catheterization; SG, significant growth; NSG, nonsignificant growth.

Two studies (47, 48) examined the diagnostic accuracy of midstream urine collection from men using either straight catheterization or suprapubic aspiration as the reference standard (Table 11). One study (47) compared midstream clean-catch specimens to those collected by suprapubic aspiration or straight catheterization in a group of hospitalized or ambulatory men, while the second study (48) compared midstream clean-catch specimens to specimens collected by suprapubic aspiration in a group of patients with spinal cord injury without indwelling catheters. Significant growth in one study (47) was defined as ≥10^4^ CFU/ml of a single or predominant species for midstream clean-catch specimens or ≥10^3^ CFU/ml for specimens collected by straight catheterization or suprapubic aspiration. Significant growth in the second study (48) was defined as any growth of ≥10^4^ CFU/ml for either collection method.

### Body-of-Evidence Qualitative Analysis

**FIG 4:**
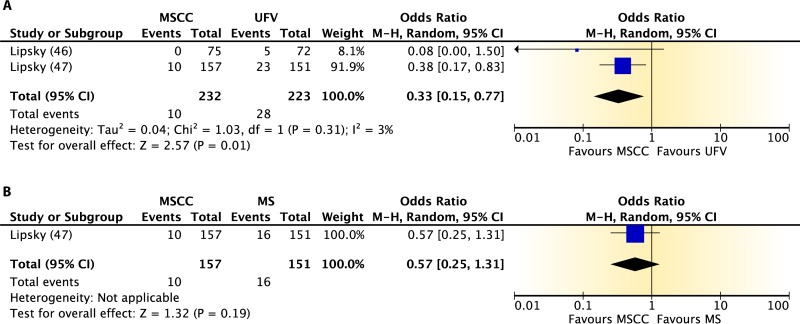
Difference in contamination levels between midstream collection with cleansing (MSCC) and first-void urine collection without cleansing (UFV) (A) or midstream collection without cleansing (MS) (B) for men.

The evidence comparing levels of contamination after midstream urine collection and uncleansed first-void collection is shown in Fig. 4A. Summary data from both studies (46, 47) found a large (77%) reduction in the odds of contamination in favor of midstream clean-catch over first-void specimens. The strength of this evidence was rated as high. Only one study (46) compared midstream collection with cleansing to midstream collection without cleansing (Fig. 4B). Results showed no difference in contamination between the two methods of collection. However, imprecision was largely due to the small event size. The diagnostic accuracy of midstream urine collection from men, with straight catheterization or suprapubic aspiration used as the reference standard, is shown in Table 12. Data for both studies found high diagnostic sensitivity (82 to 100%) and specificity (92 to 100%) for midstream clean-catch collection. However, the overall strength of this body of evidence was rated as low.

**TABLE 12:** Diagnostic accuracy of MSCC compared to SPA or CATH for the diagnosis of UTI in men^*a*^

Study (reference)	Reference standard(s)	Positivity threshold for reference test (no. of CFU/ml)	Positivity threshold for index test (no. of CFU/ml)	% sensitivity (95% CI)	% specificity (95% CI)
Lipsky et al. (46)	SPA/CATH	≥10^4^	≥10^4^	82 (67–92)	100 (92–100)
Deresinski and Perkash (48)	SPA	>10^4^	>10^4^	100 (92–100)	100 (87–100)

a MSSC, midstream clean-catch collection; SPA, suprapubic aspiration; CATH, straight catheterization. The quality rating of both studies was fair, and the index text for both was MSSC.

## COLLECTION OF URINE FROM CHILDREN

Summary information on the 14 published studies comprising the body of evidence for the clinical questions on contamination rates and the diagnostic accuracy of midstream urine collection from children is presented in Tables 13 and 14. Four studies (49–52) were given a quality rating of “good,” and 10 studies (53–62) were rated as “fair.” Six studies (49, 50, 53–56) compared differences in contamination rates in urine collected by midstream collection (with or without cleansing), collected with a sterile urine bag, or collected from diapers (Table 13). Patients studied ranged in age from 1 month to 18 years. Definitions of contamination varied among studies and included mixed growth in any concentration (54), mixed growth in any concentration or any growth of <10^5^ CFU/ml (49–51), and mixed growth at a concentration of >10^5^ CFU/ml (56); any specimen interpreted as contaminated by the clinical microbiology laboratory was also included (53).

**TABLE 13:**
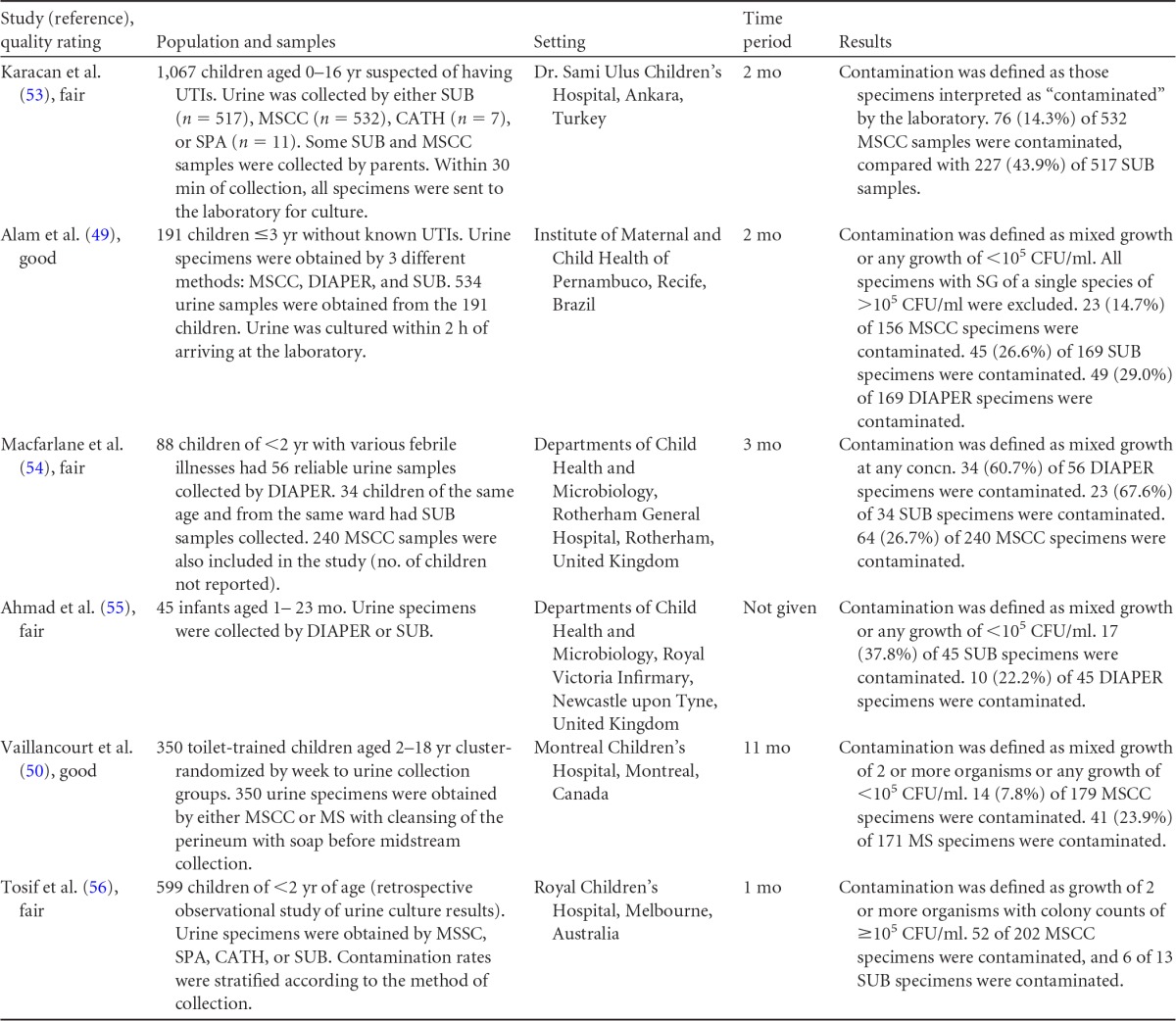
Body-of-evidence table for clinical question 7, namely, “what is the difference in contamination between MSCC, MS, SUB, and diaper collection from children?”^*a*^

a SUB, sterile urine bag collection; MSCC, midstream clean-catch collection; CATH, catheterization; SPA, suprapubic aspiration; DIAPER, diaper collection; SG, significant growth.

**TABLE 14:**
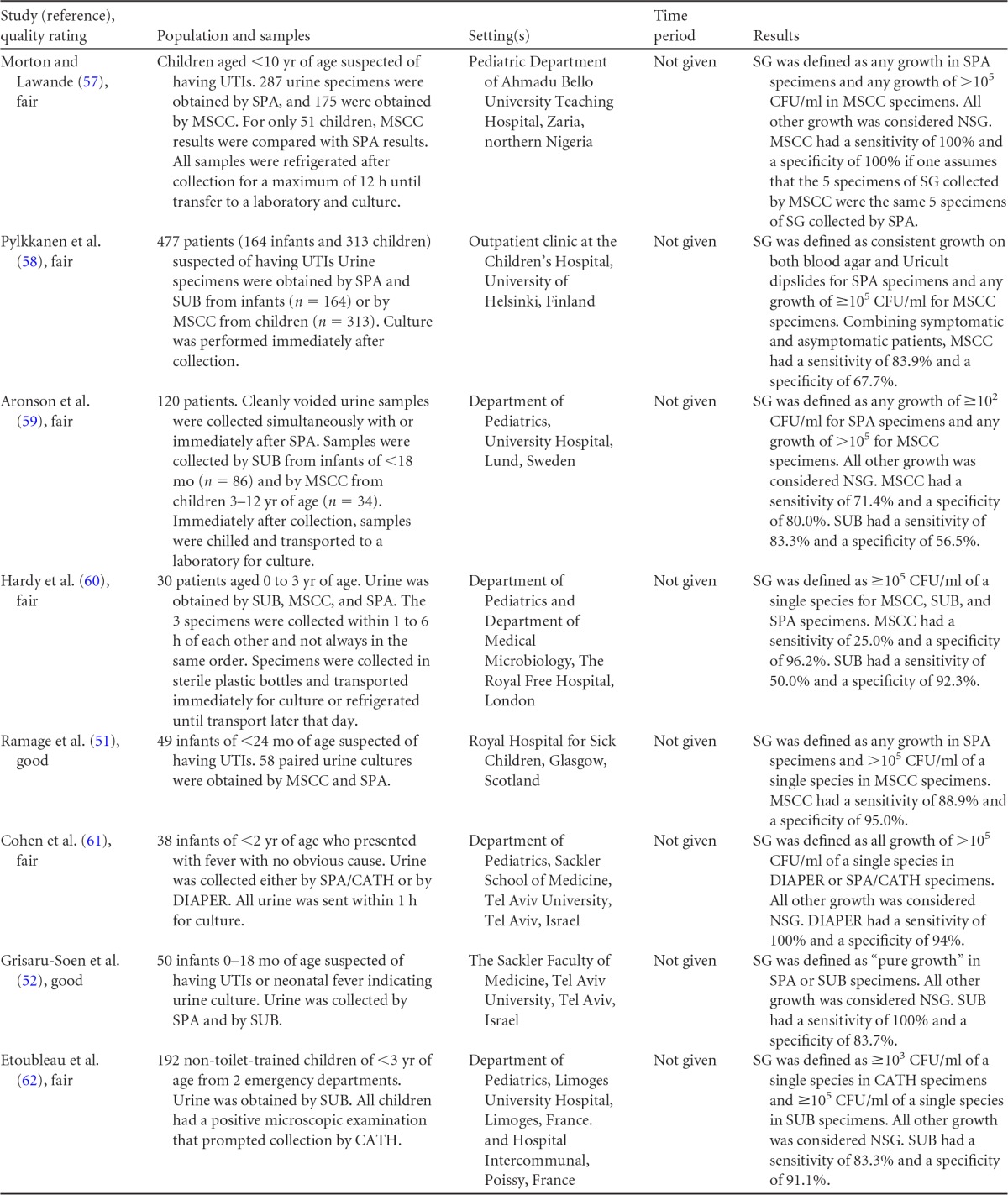
Body-of-evidence table for clinical question 8, namely, “what is the accuracy of midstream clean-catch, sterile urine bag or diaper collection compared with suprapubic aspiration or catheterization for the diagnosis of UTI in children?”^*a*^

a SUB, sterile urine bag collection; MSCC, midstream clean-catch collection; CATH, catheterization; SPA, suprapubic aspiration; DIAPER, collection from disposable diapers; SG, significant growth; NSG, nonsignificant growth.

Eight studies (51, 52, 57–62) examined the accuracy of midstream clean-catch, sterile urine bag, or diaper collection, with suprapubic aspiration or straight catheterization used as the reference standard for diagnosing urinary tract infections in children (Table 14). Patient age ranged from 0 to 10 years. Definitions of significant growth varied across studies, particularly for the reference standards. All studies except one defined significant growth for midstream clean-catch, sterile urine bag, or diaper collection as ≥10^5^ CFU/ml. The remaining study (52) defined significant growth by sterile urine bag collection as “pure growth.” Significant growth for suprapubic aspiration or straight catheterization was defined as any growth in one study (51), growth of ≥10^2^ CFU in one study (59), growth of ≥10^3^ CFU/ml in one study (62), growth of ≥10^5^ CFU/ml in three studies (57, 60, 61), and “pure growth” in one study (52). In one study (58), the definition of significant growth was unclear.

### Body-of-Evidence Qualitative Analysis

**FIG 5:**
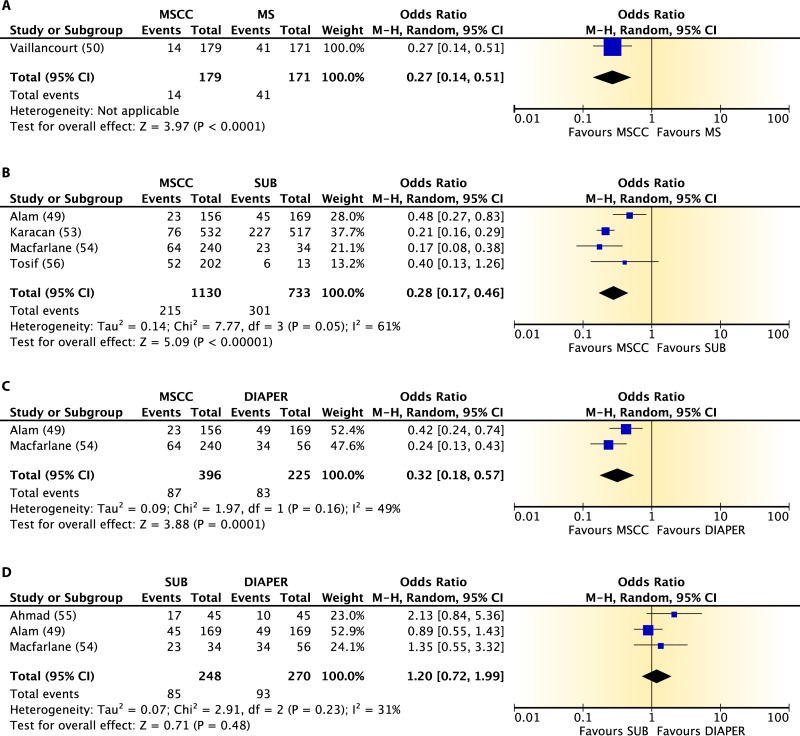
Comparative differences in contamination levels between midstream collection with cleansing (MSCC) and midstream collection without cleansing (MS) (A), midstream collection with cleansing and sterile urine bag collection (SUB) (B), midstream collection with cleansing and diaper collection (C), and sterile urine bag collection and diaper collection (D) for infants and children.

The evidence comparing contamination rates for midstream urine collection with cleansing, midstream collection without cleansing, sterile urine bag collection, and diaper collection is shown in Fig. 5. Data obtained from five observational studies (49, 53–56) and one cluster-randomized controlled trial (50) found larger reductions (68 to 73%) in the odds of contamination for specimens obtained by midstream collection with cleansing than for specimens obtained by the other methods of collection. Data from three observational studies (49, 54, 55) found no significant differences in the odds of contamination between specimens collected with sterile urine bags and specimens taken from diapers. This body of evidence was rated as high.

**FIG 6:**
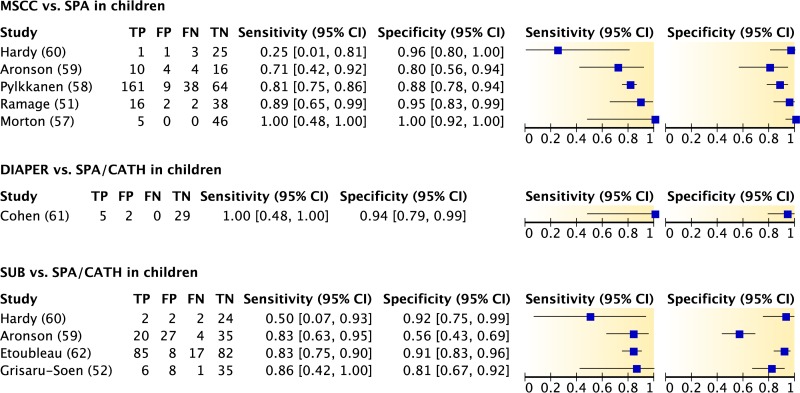
Accuracy of midstream clean-catch (MSCC), sterile urine bag (SUB), or diaper collection compared with that of suprapubic aspiration (SPA) or catheterization (CATH) for the diagnosis of urinary tract infection in children. TP, true positives; FP, false positives; FN, false negatives; TN, true negatives.

The accuracy of results for midstream clean-catch urine specimens, sterile urine bag specimens, or diaper specimens, with straight catheterization or suprapubic aspiration used as the reference standard for the diagnosis of urinary tract infection in children, is shown in Fig. 6. Data from eight observational studies showed varied results. The inability to meta-analyze the point estimates of sensitivity and specificity due to small sample and study sizes, together with heterogeneity in positivity thresholds, made interpretation difficult. Similarly, HSROC curves could not be generated, and thus it is unclear which method of noninvasive urine collection is most accurate for the diagnosis of urinary tract infection in children.

## ADDITIONAL CONSIDERATIONS

This section addresses additional considerations for evaluating preanalytical practices associated with urine cultures and the impact of these practices on contamination and diagnostic accuracy.

### Clinical Applicability

The studies included in this review reported collection, storage, and preservation of urine samples through commonly used methods for both children and adults in both inpatient and outpatient settings; results are therefore likely to apply to other health care environments. Many of the methods for collection, storage, and preservation are widely recommended (18, 63) and are typically used in most hospitals, outpatient clinics, and clinical microbiology laboratories today (21, 22). The focus of this review, however, is largely on clean-catch midstream urine collection because this method remains the most commonly used in most patient populations and settings (18). This is primarily due to its noninvasiveness; i.e., it has no risk of producing iatrogenic infection, despite the paucity of data supporting its use as a standard (63).

Controversy remains among clinical microbiologists and infectious disease physicians about the most accurate means for diagnosing urinary tract infections, including the best methods of specimen collection for women, men, children, and infants (19, 63). A recent collaboration between the American Society for Microbiology (ASM) and the Infectious Disease Societies of America (IDSA), designed to assist physicians in the appropriate use of laboratory tests for infectious diseases, addressed methods of specimen collection, as well as guidelines for testing patients for urinary tract infections (64). A recommendation was made for collection of urine in a manner to minimize contamination and included midstream collection with cleansing and immediate refrigeration of samples upon collection, although the lack of supporting data was cited (64).

In applying the findings of this review, a strength assessment of the overall body of evidence should be weighted by the quality of findings from individual reports most closely resembling populations and settings of particular interest. For instance, an overall body-of-evidence quality rating may decrease because of the aggregate number of included studies omitting study parameters of little applicability to a particular clinical setting. Researchers may take guidance, with a higher degree of confidence than the overall quality rating might indicate, from individual included studies of high or moderate strength which address specific clinical populations or settings directly comparable to their research interests. The conduct of evidence-based practice would guide clinicians to assess both the quality and the “goodness of fit” of studies relevant to their own particular questions before applying findings in support of their decisions.

This review has directed attention to the need for reexamination of preanalytic factors affecting urine culture. A great number of the studies covered in the review predate the regionalization and other significant restructuring of the delivery of microbiological services in the United States, which portend increased variation in collection, storage, and preservation methods. More studies are needed to support recommendations for specific populations, e.g., nursing facility residents needing skilled care. Important also is the growing need for documentation of health outcomes and cost-effectiveness of current practices through the implementation of well-designed, system-wide quality improvement studies. Of equal importance is the need to expand (and communicate) the literature on diagnostic testing algorithms to include nonanalytic variables, such as those measured in the included studies reported here. This systematic review provides a current and substantial literature base from which to begin investigations not only to address these gaps in current knowledge related to the effects of preanalytic factors on urine culture but also to validate these best-practice recommendations in additional settings and populations.

### Associated Harms

Methods of collecting, storing, and preserving urine specimens for the diagnosis of urinary tract infections have a critical influence on culture results. Poorly collected or preserved specimens can become easily contaminated with perineal, vaginal, and periurethral flora, which can inhibit or obscure the presence of true urinary tract pathogens. Conversely, the use of high concentrations of boric acid as a preservative has been known to inhibit urinary pathogens such as Escherichia coli and Klebsiella pneumoniae (65). Midstream urine collection may be the preferred choice for collection for most patients; however, there are patient populations and clinical scenarios where a more invasive method of collection is preferred (63). All of these issues can produce incorrect culture results, misdiagnosis, especially in asymptomatic patients, poor patient management, including the use of inappropriate or ineffective antibiotics, and potentially more complicated urinary tract infection in the long term (2, 3).

### Additional Benefits

Urine specimens that are appropriately collected, transported, stored, and preserved benefit patients by producing more-accurate culture results. In addition, such practices can provide benefit to the laboratory by allowing technologists to focus on the work-up of clinically significant pathogens rather than the growth of contaminants. Urine cultures are often a major component of the typical clinical microbiology workload (18); therefore, minimizing the processing of poor-quality urine specimens can allow the laboratory to focus its resources in a more cost-effective manner (22).

### Economic Evaluation

Proper attention to the preanalytic phase of urine cultures should decrease the number of contaminated urine specimens processed by the laboratory. It may also decrease the time it takes for microorganism identification and susceptibility testing of pathogens in infected patients by reducing the number of recollected specimens. Both of these scenarios would likely reduce health care costs for both patients and institutions by reducing the time to appropriate targeted therapy and by making more-effective use of laboratory and hospital resources. However, no economic evaluation analyses were found for the studies covered in this review.

### Feasibility of Implementation

The methods of specimen collection and handling covered in this review are feasible in all settings and patient populations and are, in fact, commonly used in most medical environments today. There are data showing the benefit of either refrigerating or chemically preserving urine samples that are not immediately processed (28–37). Furthermore, midstream urine collection, with or without cleansing, is common practice for most clinical settings and patient populations (38–62). For facilities that have historically paid little attention to the preanalytic aspects of urine culture, there may be some resistance on the part of patients and staff that is typically associated with quality improvement initiatives. Appropriate education regarding the proper collection of urine specimens may be needed for both patients and health care workers. The additional costs associated with chemical preservatives, such as boric acid, would also need to be budgeted and justified.

### Future Research Needs

The findings of this systematic review highlight the lack of recent high-quality studies that evaluate components of the preanalytical phase of urine culture. For example, the relative paucity of rigorous studies evaluating methods of storage and chemical preservation of urine specimens is troublesome considering the widespread use of these practices in many laboratories and a general consensus among microbiologists as to their benefit. A large number of the studies suffered from small sample sizes, limiting the precision of the results and reducing the likelihood that findings are applicable across a larger population. Studies also used various or unclear definitions of contamination or positivity thresholds, making meta-analysis or qualitative summary analysis problematic. Studies further suffered from missing data. For example, most studies were cross-sectional or otherwise observational (without randomization) in design, but many, particularly those retrospective in nature, did not obtain or report the results of samples from all patients obtained by all collection methods under study. These inconsistencies lead to significantly uneven comparison groups in some cases.

Future studies should strive for statistically sufficient sample sizes, use common and clearly defined definitions of contamination and thresholds for positivity, and report accuracy results across several common positivity thresholds to aid subsequent meta-analysis. An example is the number of positive/negative samples calculated if reviewers use a threshold of >10^4^ versus >10^5^ CFU per ml of urine. Studies should also be more rigorous in design, include more randomized controlled trials, and ensure paired sampling when possible in prospective or cross-sectional studies. Moreover, for all methods under evaluation, patients should have urine collected within a reasonable time frame, and the time delay between collection and culture should be clearly reported.

Finally, future studies should strive to obtain data on downstream patient-centered outcomes as influenced by different methods of collection, preservation, or storage of urine that are under evaluation. This broader measurement pool includes system-oriented outcomes, such as time to targeted therapy, cost of antibiotic use, number of UTI discharge diagnoses, or number of Clostridium difficile cases avoided, such that the direct or indirect impact of implementing a particular preanalytic practice can be measured at the patient and organizational level. Information provided in Appendix 2 can be used as a guide to organize and plan studies as well as collect data for any quality improvement project that examines preanalytical practices associated with urine cultures.

### Limitations

The LMBP systematic review method is compatible with other standards of practice for systematic reviews (24) but includes some unique elements, such as the rating of study quality. Rating study quality is based on attributes such as facility description, study setting and design, practice description, outcome measures, and results. How studies are ultimately considered for inclusion in the review depends on consensus assessments that may be influenced by such things as a rater's professional background and experience. Indeed, several on-topic studies were excluded because of limitations identified during quality evaluation, mostly related to poor reporting of important study, practice, or outcome details. This is likely somewhat explained by the publication dates of many of the studies, with several of both the excluded and included studies having been published in the late 1960s. As is the case with most systematic reviews, attempts were made to limit publication bias by soliciting unpublished data; however, no unpublished data were submitted. Moreover, restricting the review to English-language studies may also introduce bias.

Outside the limitations of the review process, there were a number of limitations in this review that affected the ability to draw firm conclusions and make recommendations. Most of these limitations were addressed above in the context of future research, but additional limitations will be discussed here. The study settings varied across included studies. Both inpatient and outpatient settings were included, and the specific setting examined in each study—emergency department, adolescent clinic, obstetric clinic, etc.—may not be generalizable to other settings. Some settings may be better equipped to perform certain collection methods or to educate patients or parents on how to perform certain collection methods. Similarly, the patient populations under study varied. Some studies included healthy asymptomatic patients, while others included patients with more-severe conditions, such as spinal cord injury patients. This too might affect the generalizability of results. Within the body of evidence for children, studies often included patients ranging in age from 0 to 16 years. Unfortunately, there were not enough data available to properly stratify children, such as infants, into smaller age groups, and because of this, results may not be generalizable to patients of a specific age. Finally, as discussed above, an important limitation was the variability in positivity thresholds and definitions of contamination used across studies. Although several guidelines have been developed to address definitions of significant bacteriuria for culture (18, 63, 66–68), these guidelines are not always consistent, and this lack of consistency is reflected in the studies and results reported in this review.

## CONCLUSIONS AND RECOMMENDATIONS

A summary of the findings of this evidence-based review of urine culture preanalytics can be found in Table 15. Conclusions are categorized as “recommended,” “not recommended,” or “no recommendation for or against” and refer to studies of urine collected by noninvasive methods:

1. No recommendation for or against is made for delayed processing of urine that is stored at room temperature, refrigerated, or preserved in boric acid due to insufficient evidence. Data from nine studies receiving a “fair” quality rating suggest that both refrigeration and boric acid adequately preserve urine specimens for up to 24 h prior to their being processed. Furthermore, data from three studies receiving a “fair” quality rating suggest that urine held at room temperature for more than 4 h should not be processed due to overgrowth of both clinically significant and contaminating flora. However, because the overall strength of the body of evidence was rated as low, no recommendation for or against can be made due to insufficient evidence. This does not preclude the use of refrigeration or chemical preservatives in clinical practice. It does indicate, however, that more systematic studies evaluating the utility of these measures are needed.
2. If noninvasive collection is being considered for women, midstream collection with cleansing is recommended, but no recommendation for or against is made for midstream collection without cleansing due to insufficient evidence. Data from two studies, including one randomized controlled trial receiving a “good” quality rating and three studies receiving a “fair” quality rating, show that contamination rates are similar between specimens obtained by midstream collection with and without cleansing. The overall strength of this body of evidence was rated as high. However, whether midstream collection can be routinely used in place of straight catheterization is unclear. Data from three studies, two with a quality rating of “fair” and one with a rating of “good,” suggest that clean-catch midstream urine collection is highly accurate for diagnosing urinary tract infections in women; however, because the overall strength of this body of evidence was rated as low, no recommendation for or against can be made.
3. If noninvasive collection is being considered for men, midstream collection with cleansing is recommended and collection of first-void urine is not recommended. No recommendation for or against is made for collection of midstream urine without cleansing due to insufficient evidence. Data from two studies, one with a quality rating of “good” and one with a rating of “fair,” found a large reduction in the level of contamination in specimens obtained by midstream collection with cleansing compared to the level of contamination after collection of first-void urine. This body of evidence was rated as high. Although data from one study rated as “good” quality found no difference in contamination between midstream urine collected with and that collected without cleansing, imprecision was large due to the small event size, and no recommendation can be made as to which method is superior. Whether midstream collection can be used routinely in place of straight catheterization or suprapubic aspiration is unclear. Data from two studies receiving a “fair” quality rating suggest that midstream collection with cleansing is highly accurate for the diagnosis of urinary tract infections in men; however, because the overall strength of the body of evidence was rated as low, no recommendation for or against can be made.
4. If noninvasive collection is being considered for children, midstream collection with cleansing is recommended and collection with sterile urine bags, from diapers, or midstream without cleansing is not recommended. Data from six studies, two with a quality rating of “good” and four rated as “fair,” found large reductions in contamination in midstream clean-catch urine specimens compared to contamination after other noninvasive methods of collection. This body of evidence was rated as high. Whether midstream collection with cleansing can be routinely used in place of catheterization or suprapubic aspiration is unclear. Data from eight studies, two with a quality rating of “good” and six rated as “fair,” suggest that midstream collection with cleansing is accurate for the diagnosis of urinary tract infections in infants and children and that midstream collection with cleansing has higher average accuracy than sterile urine bag collection (data for diaper collection was lacking). However, the overall strength of evidence was low, as multivariate modeling could not be performed; thus, no recommendation for or against can be made due to insufficient evidence.

**TABLE 15:** Summary of findings of our evidence-based review of urine culture preanalytics

Quality issue	Body of evidence quality	Body of evidence strength	Recommended	Not recommended	No recommendation for or against due to insufficient evidence
Delayed processing of urine stored at room temp vs refrigeration vs boric acid	Fair	Low			**×**
Midstream urine collection from women with cleansing	Good/fair	High	**×**		
Midstream urine collection from women without cleansing	Fair	Low			**×**
Midstream urine collection from women vs collection by straight catheterization	Fair/good	Low			**×**
Midstream urine collection from men with cleansing	Good/fair	High	**×**		
Midstream urine collection from men without cleansing	Fair	Low			**×**
First-void urine collection from men	Good/fair	High		**×**	
Midstream urine collection from men vs collection by straight catheterization or suprapubic aspiration	Fair	Low			**×**
Midstream urine collection from children with cleansing	Fair/good	High	**×**		
Midstream urine collection from children without cleansing	Fair/good	High		**×**	
Urine collection from children with sterile urine bags and/or from diapers	Fair/good	High		**×**	
Midstream urine collection from children vs collection by straight catheterization or suprapubic aspiration	Fair/good	Low			**×**

## APPENDIX 1

The members of the Laboratory Medicine Best Practices Workgroup from 2012 to 2014 were as follows: Robert H. Christenson, University of Maryland Medical Center, Baltimore, MD; John Fontanesi, UC—San Diego Medical School, La Jolla, CA; Julie Gayken, Regions Hospital, St. Paul, MN; James Nichols, Vanderbilt University Medical Center, Nashville, TN; Mary Nix, Agency for Healthcare Research and Quality, Rockville, MD; Milenko Tanasijevic, Brigham and Women's Hospital, Boston, MA; Sharon Geaghan, Stanford, University School of Medicine, Stanford, CA; Christine Litwin, Georgia Health Sciences University, Augusta, GA; Thomas Lorey, Permanente Medical Group Regional Laboratory, Richmond, CA; Bernadette Mazurek Melnyk, The Ohio State University, Columbus, OH; Anton Piskac, Methodist Health System, Omaha, NE; Jennifer Rhamy, St. Mary's Hospital, Oakbrook Terrace, IL; Christopher Lee Roy, Brigham and Women's Hospital, Boston, MA; and Melissa Singer (*ex officio*), Centers for Medicare and Medicaid Services, Baltimore, MD.

## APPENDIX 2

### LMBP Evaluation of Preanalytic Practices for the Contamination and Diagnostic Accuracy of Urine Cultures

#### Suggested guidance for future studies.

**FIG A1:**
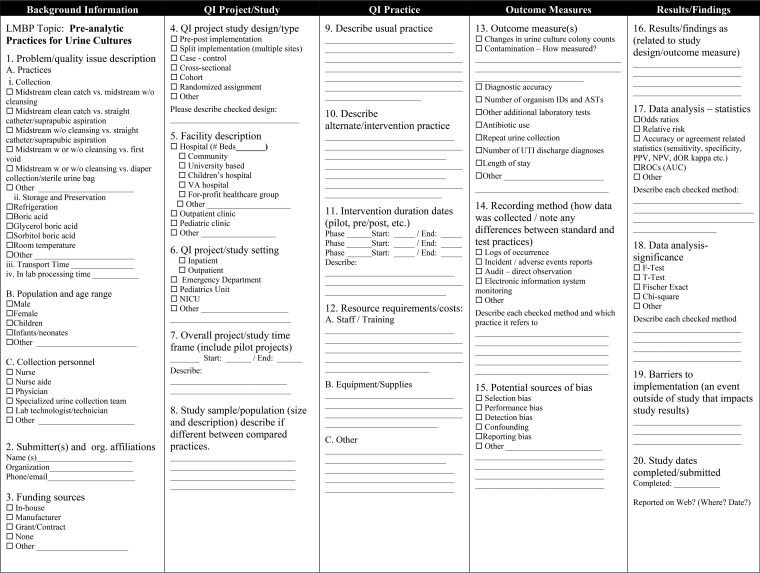
Form for use in collecting data for any QI project that examines preanalytical practices associated with urine cultures.

This review identified and rated practices associated with the collection, preservation, and storage of urine specimens for culture and assessed the impact of these preanalytic practices on the diagnostic accuracy of urine culture microbiology. In theory, the design, description, methods, data collection, and analysis for any study should be written and documented so that other investigators can reproduce exactly the same study in their laboratory, with their results validating or verifying those of the original study. The following organizational plan with instructions can be used as a guide for quality improvement project design, implementation, and evaluation of preanalytic practices associated with urine cultures. Figure A1 shows a form for use in collecting data for any QI project that examines preanalytical practices associated with urine cultures.

### Background Information

1. Problem/quality issue description.
  - Practices and equipment. Describe what preanalytic practices associated with the collection and preservation of urine for culture were studied and exactly how specimens were collected. Include examples of educational material handed to patients or displayed on walls in areas where patients were seen.
    - Collection. Indicate whether specimens were obtained by midstream clean-catch collection versus midstream collection without cleansing, midstream clean-catch collection versus straight catheter collection and/or suprapubic aspiration, midstream collection without cleansing versus straight catheter collection and/or suprapubic aspiration, midstream collection with or without cleansing versus first-void urine collection, midstream collection with or without cleansing versus diaper collection and/or sterile urine bag collection, or other means.
    - Preservation/storage/transport. Include the time from collection of the specimen to the addition of preservative and how long it took the specimen to reach the lab after collection, as well as how long it took from receipt in the lab to setup of culture. Indicate whether boric acid, glycerol boric acid, or sorbitol boric acid was used as a preservative, whether the specimen was stored in a refrigerator or at room temperature, and any other relevant preservation or storage information.
  - Population under study and age ranges. Include physical differences which may affect the collection of the specimen, such as physical disability, the presence of a foreskin in males, the presence of diapers, etc. With infants and neonates, consider tighter age ranges, such as 0 to 2, 2 to 4, etc.
  - Collection personnel. Indicate whether the specimen was collected by a nurse, nurse's aide, physician, specialized urine collection team, lab technologist or technician, or other staff member.
2. Submitter(s) and organization affiliation. For additional questions concerning the quality improvement (QI) study, contact information is required.
3. Funding source(s). Refer to the chart in Fig. A1. Check all boxes that apply.

### QI Project/Study

4. QI project study design/type. With similar patient populations, describe the methods/approaches used for your project with regard to age, sex, ethnicity, and/or diagnosis to limit bias.
  - Pre- and postimplementation. Observations are made before and after the implementation of an intervention.
  - Split implementation design. Indicate whether multiple sites were used to conduct the QI study.
  - Case-control study. Indicate whether the study compared subjects with a specific outcome of interest (cases) with subjects from the same source population but without that outcome (controls) to examine the association between the outcome and prior exposure (for which there was an intervention).
  - Cross-sectional associations. Collect information on interventions (past or present) and current health outcomes, i.e., those that are restricted to health states, for a group of people at a particular point in time, to examine associations between the outcomes and exposure to interventions.
  - Cohort. A defined group of people (the cohort) is followed over time to examine associations between different interventions received and subsequent outcomes.
  - Randomized assignment. Patients are randomly selected to receive the intervention practice or the comparator practice.
  - Other study design used in this QI project. Describe the study design selected.
5. Facility description. Provide a complete description of the facility type and the number of beds (or patients if the facility is an outpatient facility).
6. Study setting. Select the unit(s) within the facility where the practice was implemented, e.g., inpatient, outpatient, emergency department, pediatric unit, neonatal intensive-care unit, or other.
7. Overall project/study time frame. Record the start and end dates for the new and usual practices; if pilot testing was conducted, include start/end dates for pilot testing of the new practice. Note that this is not the same as the QI study period but rather the dates during which these practices were being used in the unit(s) in which the study was done. Put “NA” If some dates are not available.
8. Sampling strategy. The sample size is the number of patients/observations used for the usual (current) practice and the alternate practice. Use the largest available sample size at each time of measurement. For results to be reliable, the implemented practice should be the only thing affecting the results. It is the largest sample size available that represents only the results of the usual practice and the largest sample size that represents only the results of the alternate practice. Describe your sample set (tests, patient specimens, patients, or type of patient specimens) and the sample size (example: prior to [usual] practice [15,000 patient specimens tested] and after [alternate] practice [13,200 patient specimens tested]).

Optimally, a power analysis should be performed prior to confirmation of sample size. Statistical power is the probability of concluding that there is a difference when there is, in fact, a difference between your standard method and your new method (i.e., the probability that your study will detect a difference, given that one truly exists). An example of a nomogram for sample size calculation can be found in reference 69.

Statistical power is the probability of concluding that there is a true and significant difference between your comparator and intervention, thus minimizing type I and type II errors (sensitivity and specificity).

For sample description, refer to the following list.

- Random sampling. Subjects (patients) are selected for study inclusion using a formal random selection process applied to the census.
- Convenience sampling. Some subset of the census is selected since it is easy to access. For example, using only data from records of patients whom you can easily reach would be a convenience sample.
- Census sampling. All participants within a specified time period or location are used in census sampling.
- Other. Describe whether you are using a different sampling method. If you are using anything other than random sampling, convenience sampling, or census sampling, you need a statistician to identify sampling strategy.

### QI Practice

9. Describe the original (usual) practice. Describe the original (usual) practice(s) that will be compared to the new practice/policy/technology implemented.
10. Describe the alternate/intervention (new) practice. Describe the new practice/policy/technology, including the characteristics and components for ongoing day-to-day operations.
11. Intervention duration dates (pilot, pre/postintervention, etc.). Record the start and end dates for the pilot testing, usual practice, and new practice. This is the date on which the particular QI project was implemented and the date on which it ended. Note that a pilot test may not have been used in this study. There should be no gaps in the QI project data collection once it begins.
12. Resource requirements/costs. Describe the requirements and costs for starting and sustaining the new practice during the study. If this information is not available, list “not known.” Do not list the cost of the practice that is currently being used to do patient testing.

### Outcome Measures

13. Outcome measure(s). Describe how the impact of the practice was measured. Provide the specific outcome(s) and corresponding specifications/definitions used to assess or track the impact of the practices implemented. An example is a description of how urine culture contamination rates were affected or how they had an impact on the diagnostic accuracy of urine culture.
14. Recording method. Describe each method used to collect data and to which practice (usual or new) it refers.
15. Potential sources of bias. Bias is the tendency to produce results that depart systematically from the “true” results. Bias is any nonrandom factor in the conduct of a study that can influence the results of a study.
  - Selection bias. Selection bias occurs when studies are conditioned on (that is, they differentially select for) common effects of the exposure and the outcome. Selection bias occurs after exposure and arises when the associations between exposure and outcome are different for those who participate and those who do not participate in a study (i.e., all those who are theoretically eligible). This bias includes inappropriate selection of controls in a case-control study, different losses to follow-up for groups being compared (attrition bias), incidence/prevalence bias, nonresponse bias, and inclusion or exclusion of specific groups for study.
  - Performance bias. Performance bias includes systematic differences in the types of care provided to participants and protocol deviations. Examples include contamination of the control group with the exposure or intervention, unbalanced provision of additional interventions or cointerventions, a difference in cointerventions, and providers and participants not being adequately blind to the study results.
  - Detection bias. Detection bias includes systematic differences in outcome assessments among groups being compared. Reasons for this bias include misclassification of the exposure, intervention, covariates, or outcomes because of varying definitions, timings, diagnostic thresholds, and memories of an event; assessors not being adequately blind to the study results; and faulty measurement techniques. Erroneous statistical analysis might also affect the validity of effect estimates.
  - Confounding bias. Confounding bias is the presence of systematic differences between baseline characteristics of the groups that arise when patient prognostic characteristics, such as disease severity or comorbidity, influence both treatment source and outcomes. Confounders are the common cause for intervention and exposure; they occur before exposure. Confounding by indication can occur from self-selection of treatments or physician-directed selection of treatments.
  - Reporting bias. Reporting bias is the presence of systematic differences between reported and unreported findings (e.g., differential reporting of outcomes or harms, incomplete reporting of study findings, and potential for bias in reporting through source of funding).

### Results/Findings

16. Results/findings as related to study design/outcome measure. For each outcome provided, summarize the results/findings of the study/project related to the practice. Provide the total number of observations (samples) on which the results are based and statistical tests results, if a statistical analysis was performed. Include findings related to cost savings or shortened length of stay, if applicable.
17. Data analysis with regard to statistics. Describe the statistic used to measure the strength of association or the statistical measures of the performance of classification tests (e.g., sensitivity, specificity). Examples are as follows.
  - With first-void urine collection, 10 urine samples are contaminated per 100 urine cultures performed (10% contamination rate).
  - With midstream clean-catch urine collection, 3 urine samples are contaminated per 100 urine cultures performed (3% contamination rate).
  - The odds ratio is 0.28 (3/10/97/90).
18. Data analysis with regard to significance. Describe the tests of significance. Include calculations of the statistical significance of a difference between the usual practice and the alternate practice on the measured outcomes listed in item 14.
19. Barriers to implementation. Describe any outside activities occurring at the same time as the project, such as staff changes or new policy, that may have influenced the results of the project. Describe any barriers that directly impacted the project.
20. Study dates completed or report submitted. Dates should include the date that the study was completed, the date it was reported (and where it was reported), and the date it was submitted to the LMBP initiative.

## APPENDIX 3

Refer to Tables A1 to A4 for evidence summaries of results for storage (refrigeration versus room temperature) and boric acid preservation of urine, contamination and diagnostic accuracy of urine collected from women, contamination and diagnostic accuracy of urine collected from men, and contamination and diagnostic accuracy of urine collected from children.

**TABLE A1:**
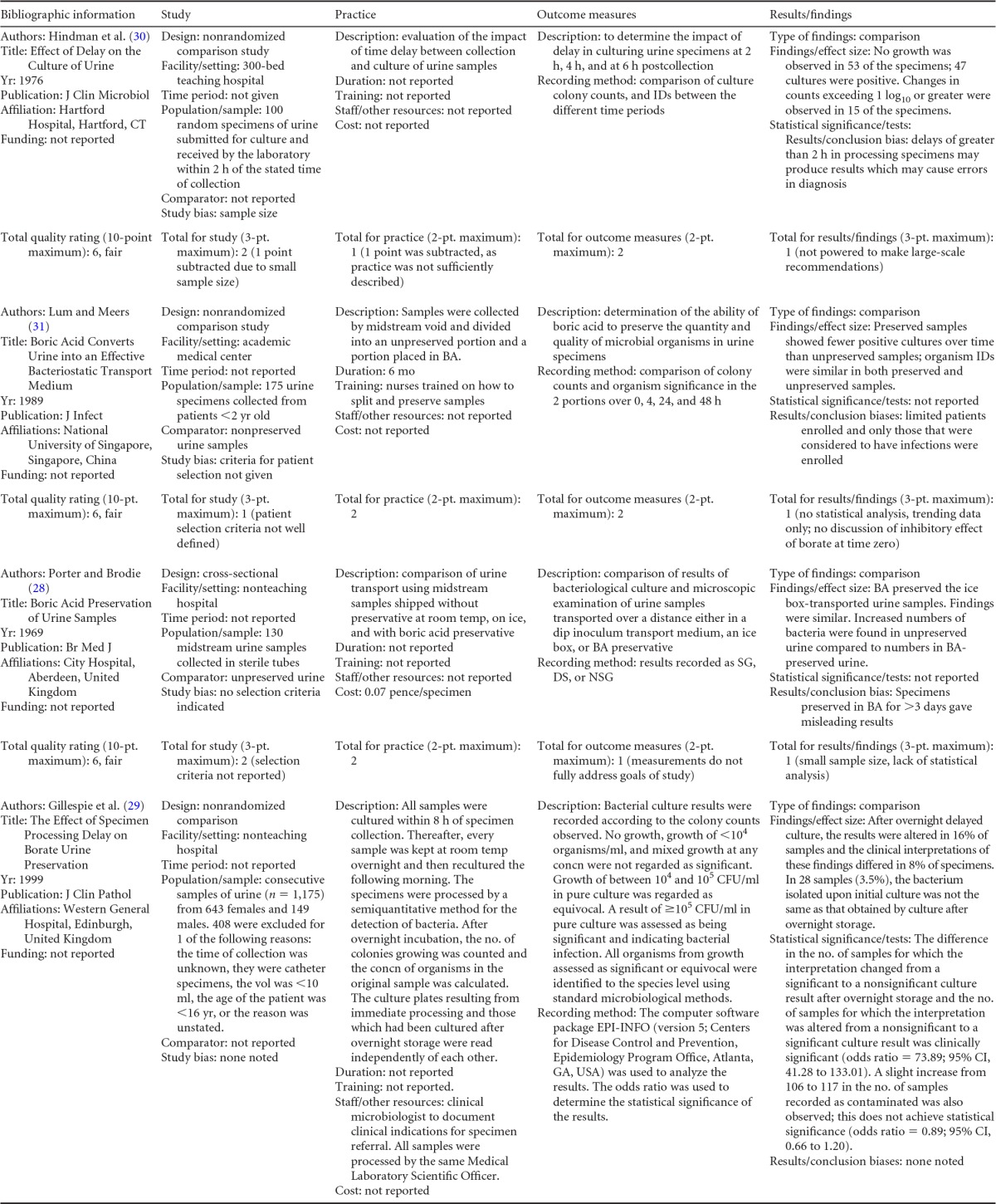

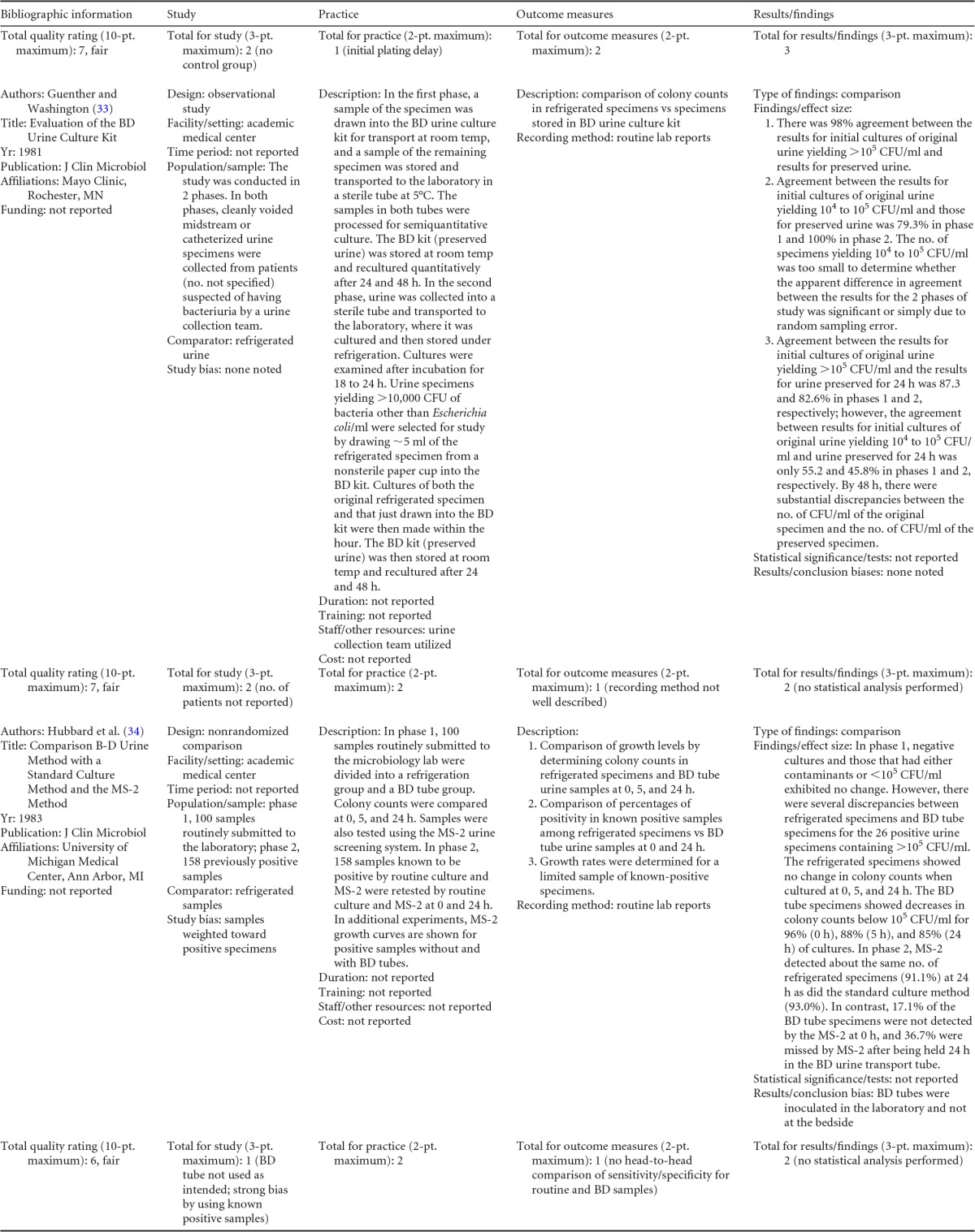

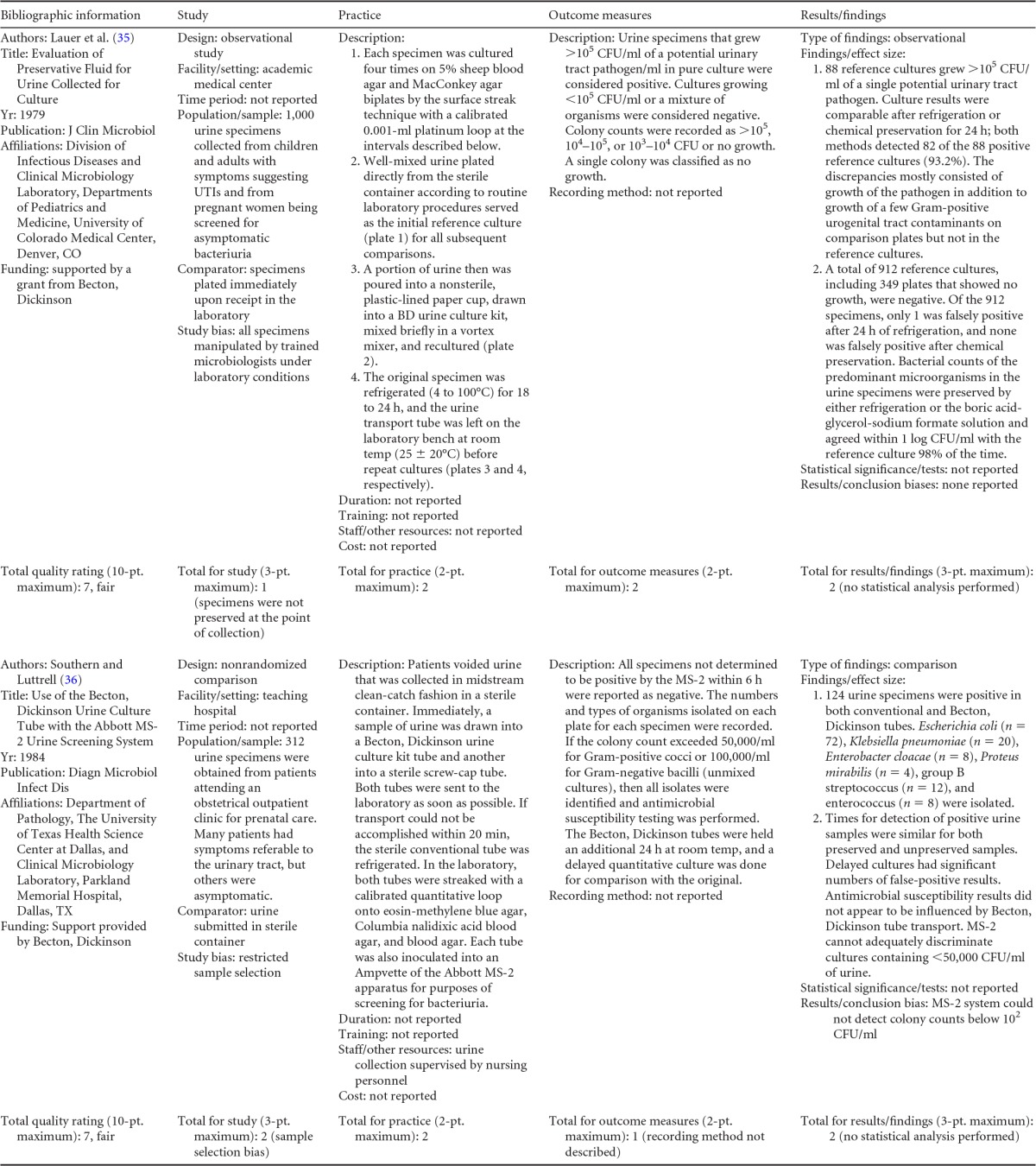

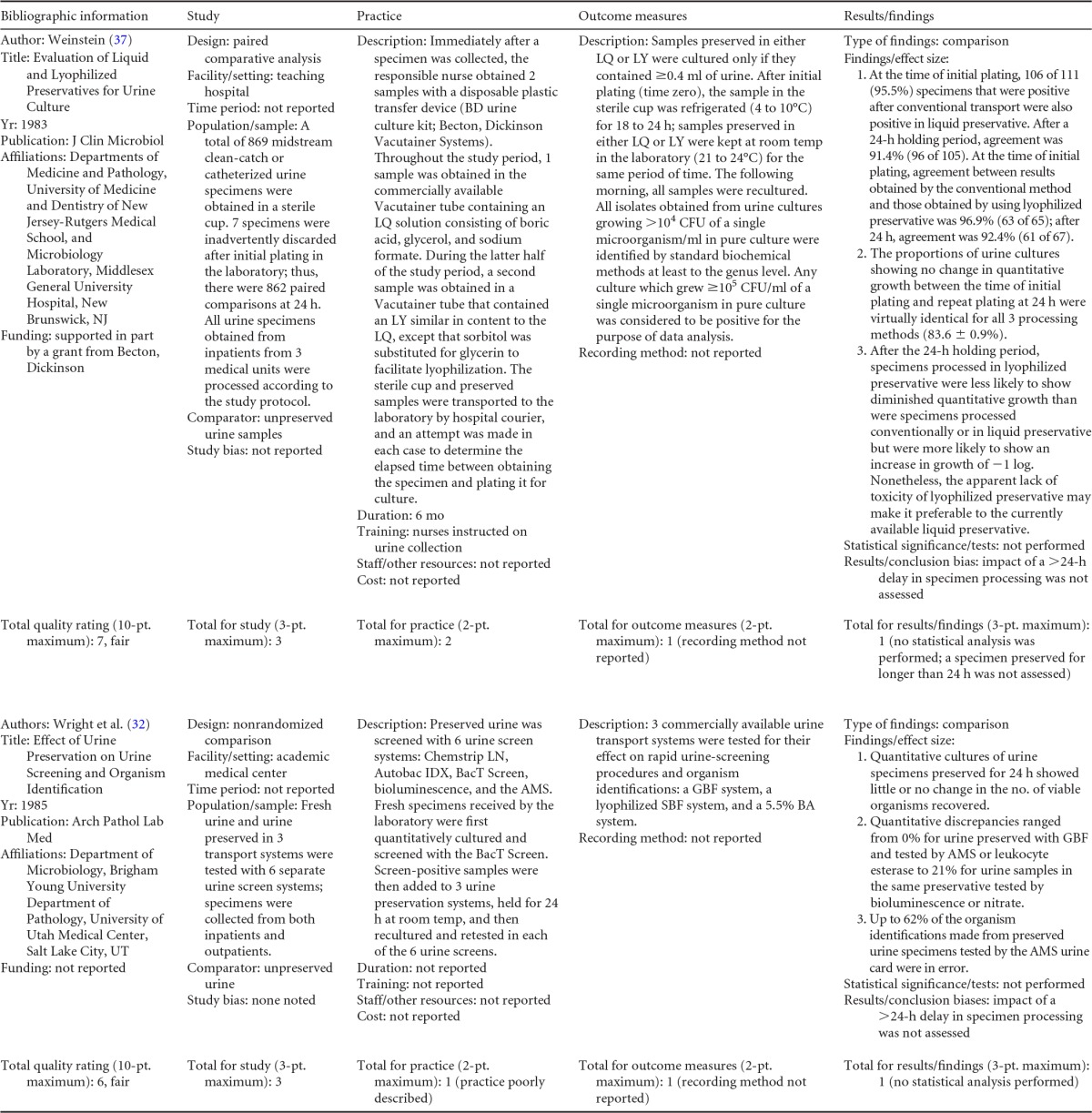
Evidence summary table for storage (refrigeration versus room temperature) and boric acid preservation of urine^*a*^

a For scoring information, see Christenson et al. (24). IDs, identifications; pt., point; 95% CI, 95% confidence interval; SG, significant growth; DSG, doubtful significant growth; NSG, nonsignificant growth; LQ, liquid preservative; LY, lyophilized preservative; AMS, Automicrobic system; GBF, glycerin-boric acid-sodium formate; SBF, sorbitol-boric acid-sodium formate; BA, boric acid.

**TABLE A2:**
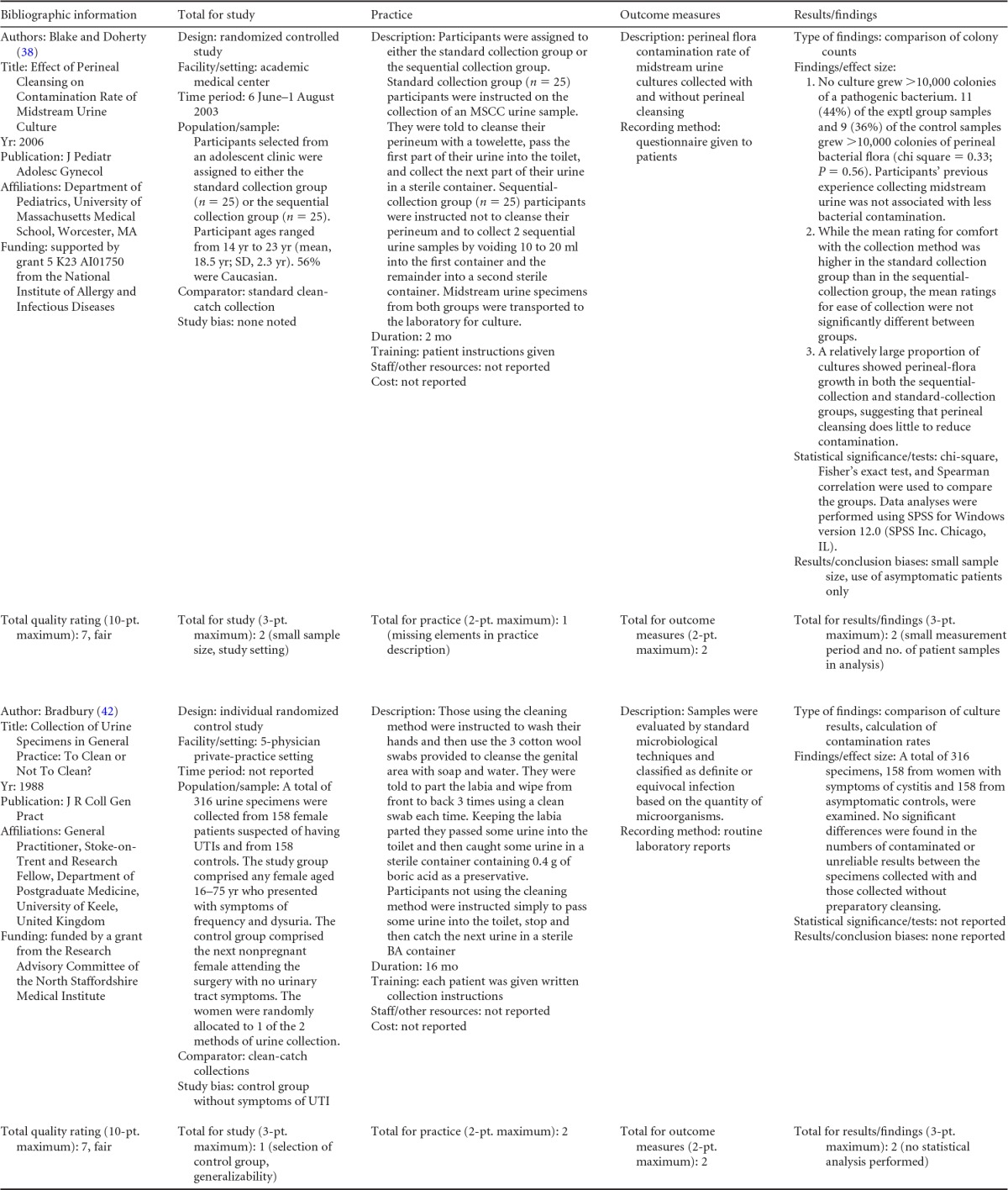

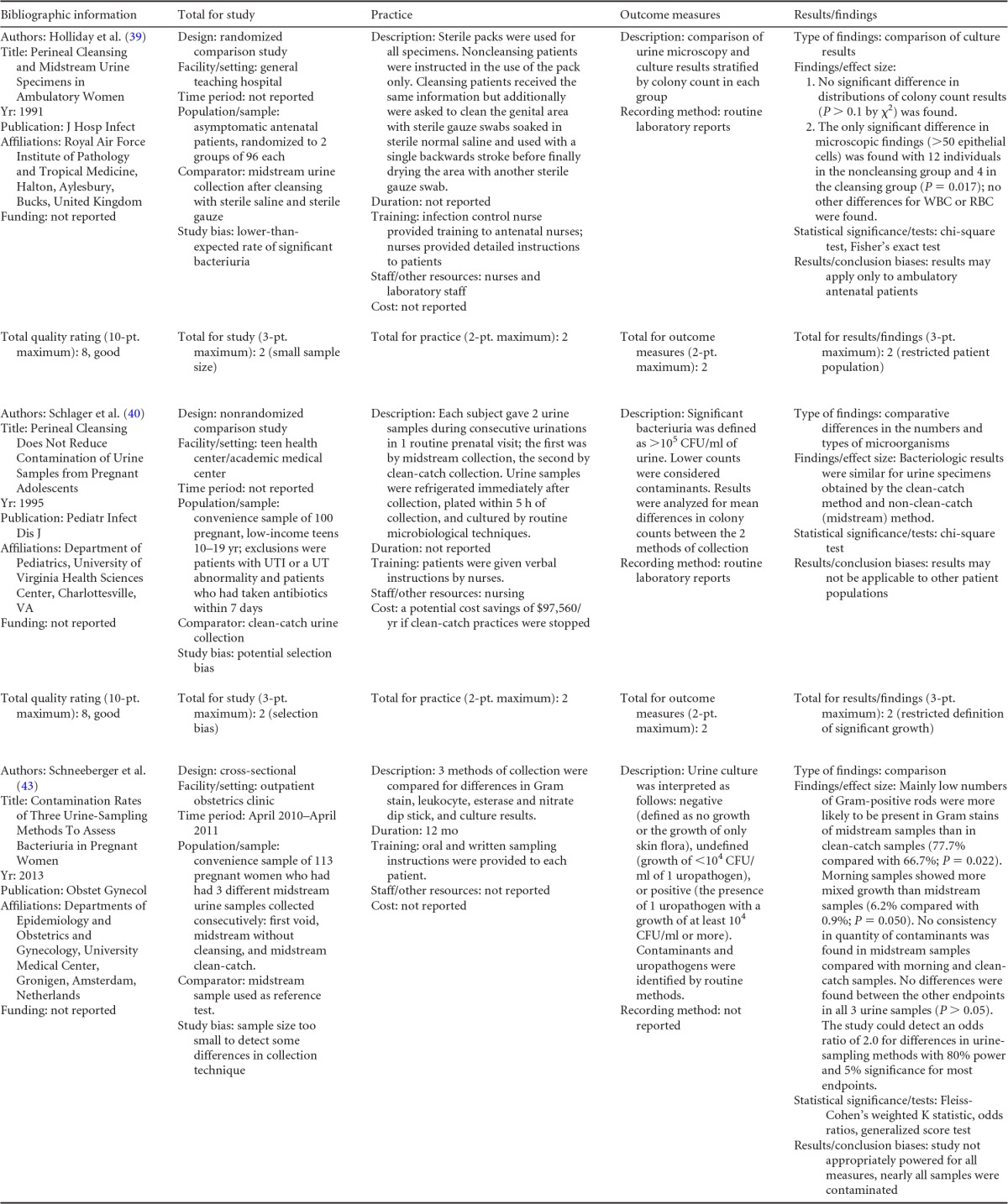

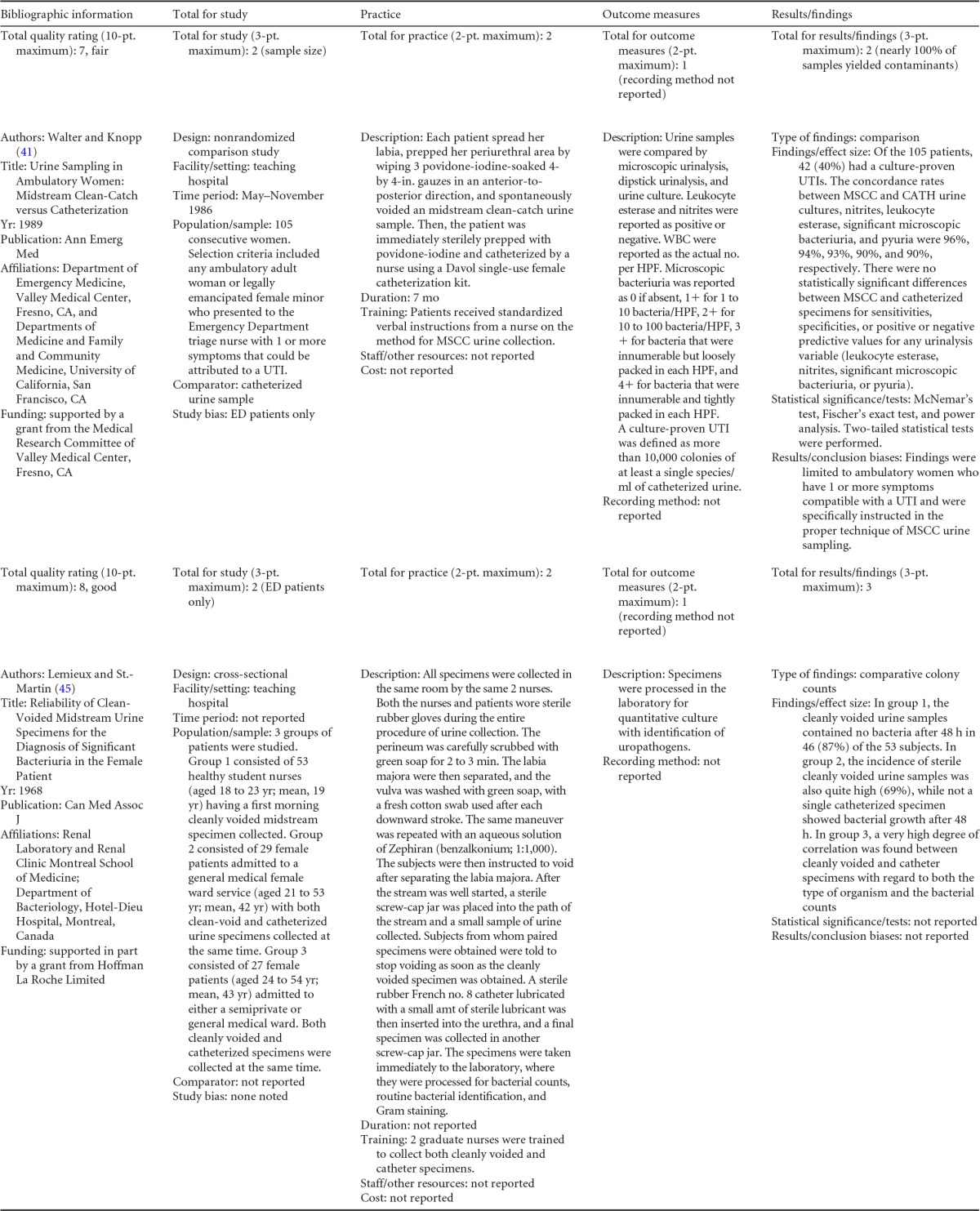

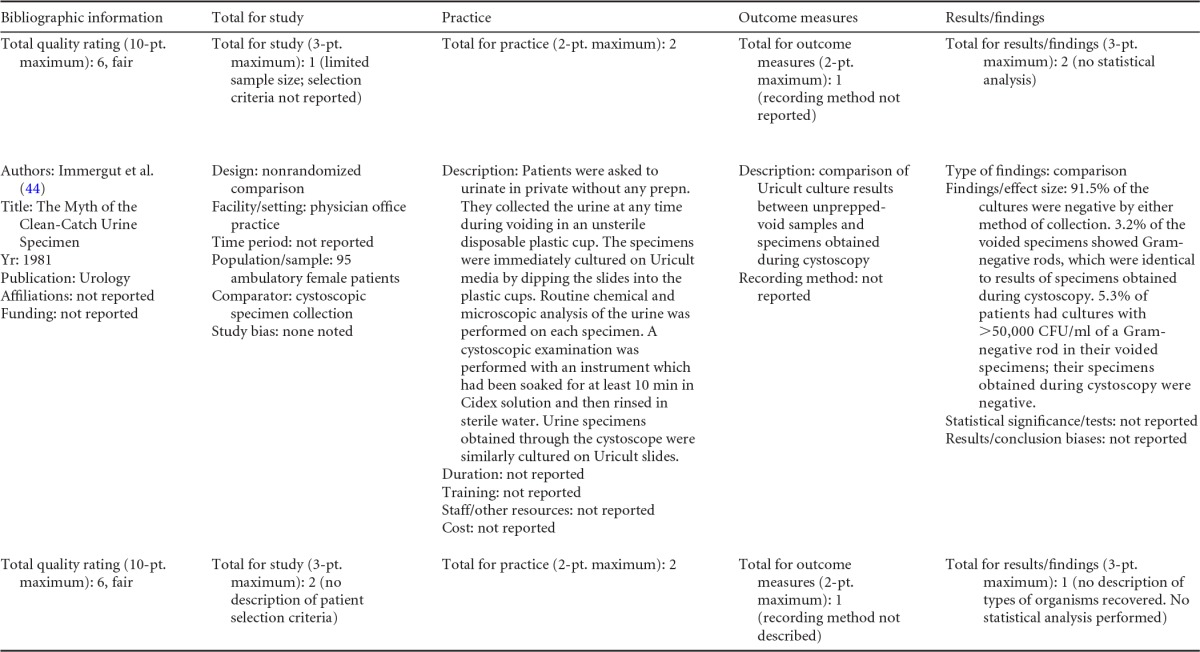
Evidence summary tables for contamination and diagnostic accuracy of urine collected from women^*a*^

a MSCC, midstream clean-catch collection; CATH, catheterization; HPF, high-power field.

**TABLE A3:**
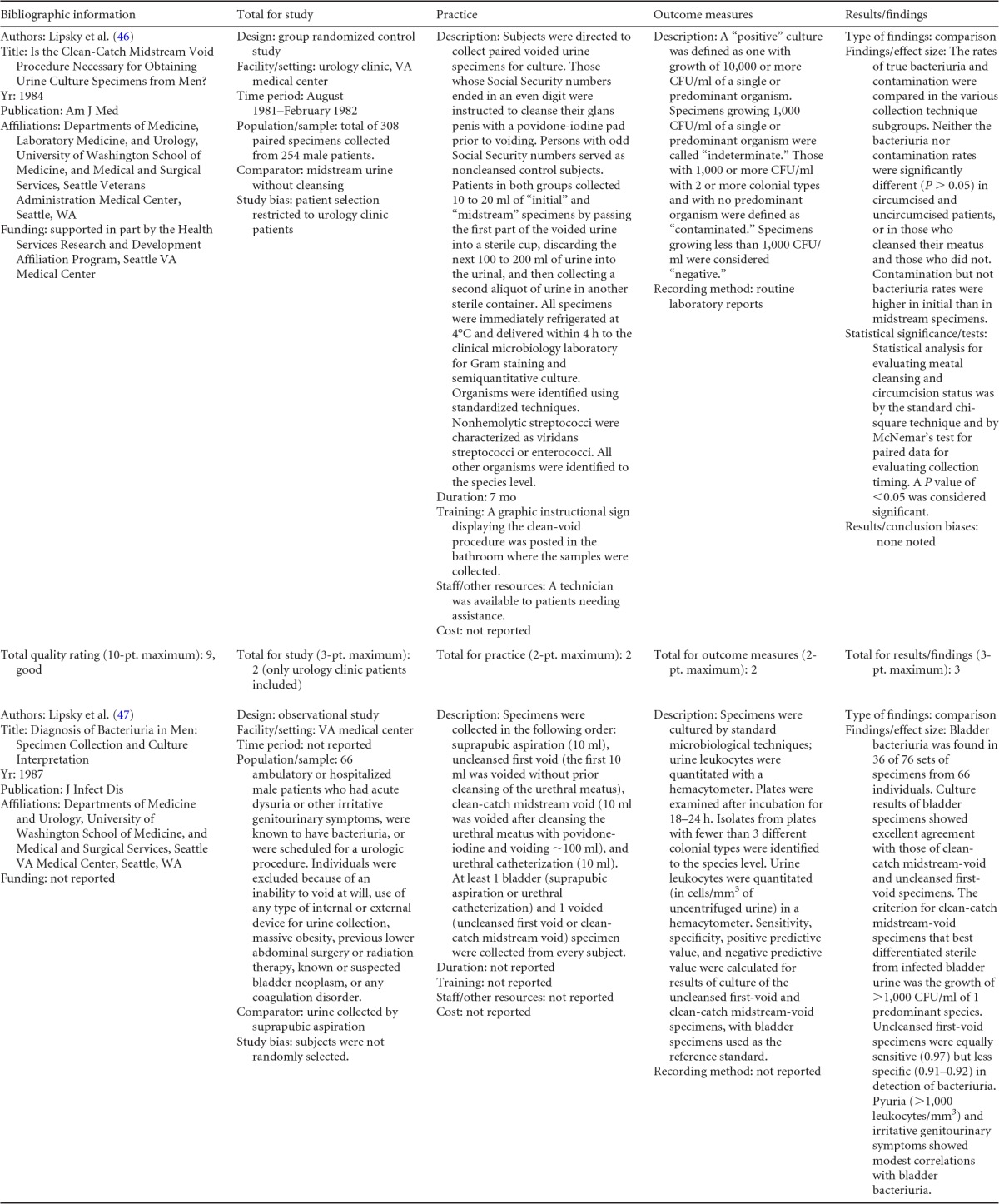

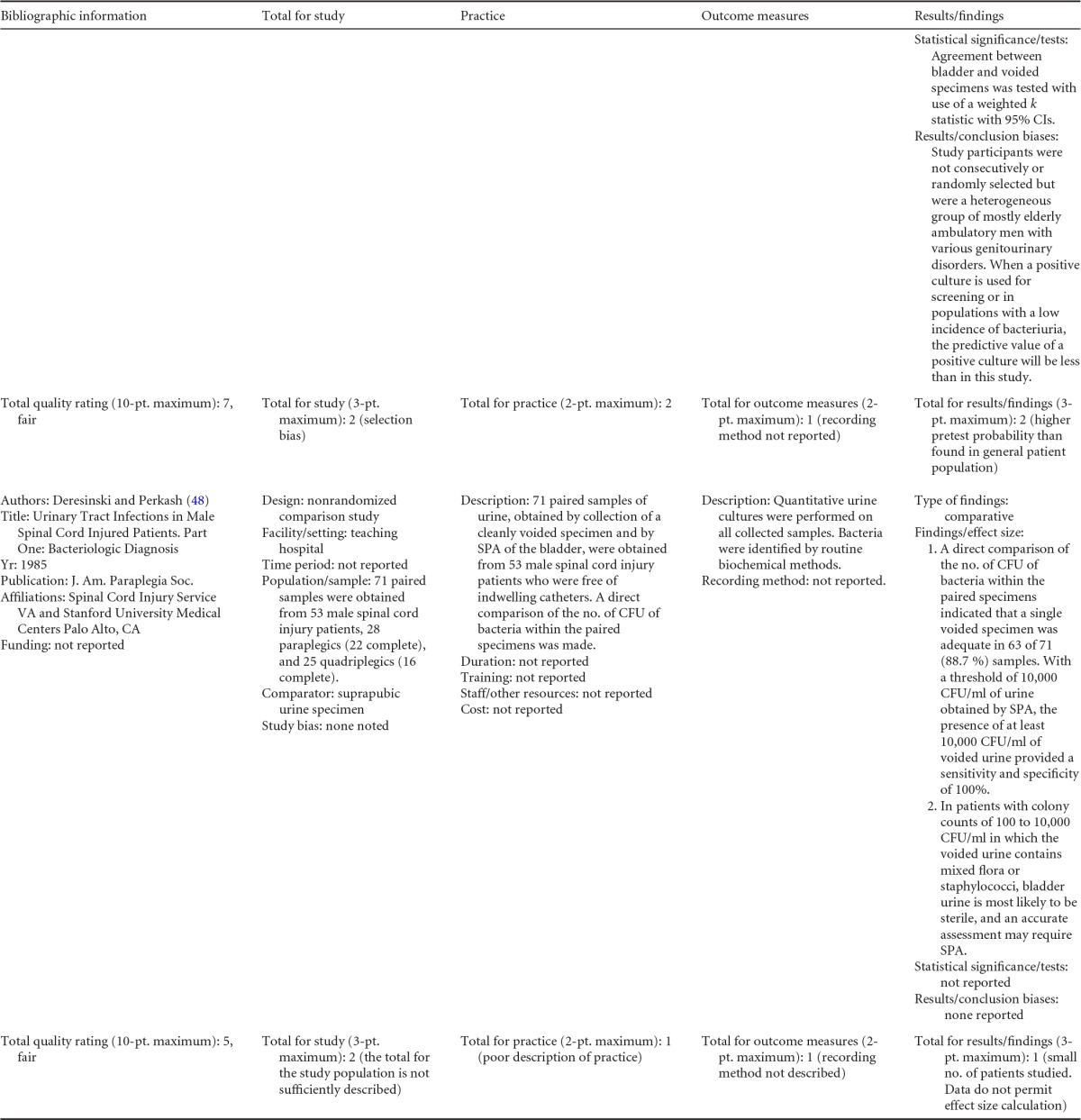
Evidence summary table for contamination and diagnostic accuracy of urine collected from men^*a*^

a SPA, suprapubic aspiration.

**TABLE A4:**
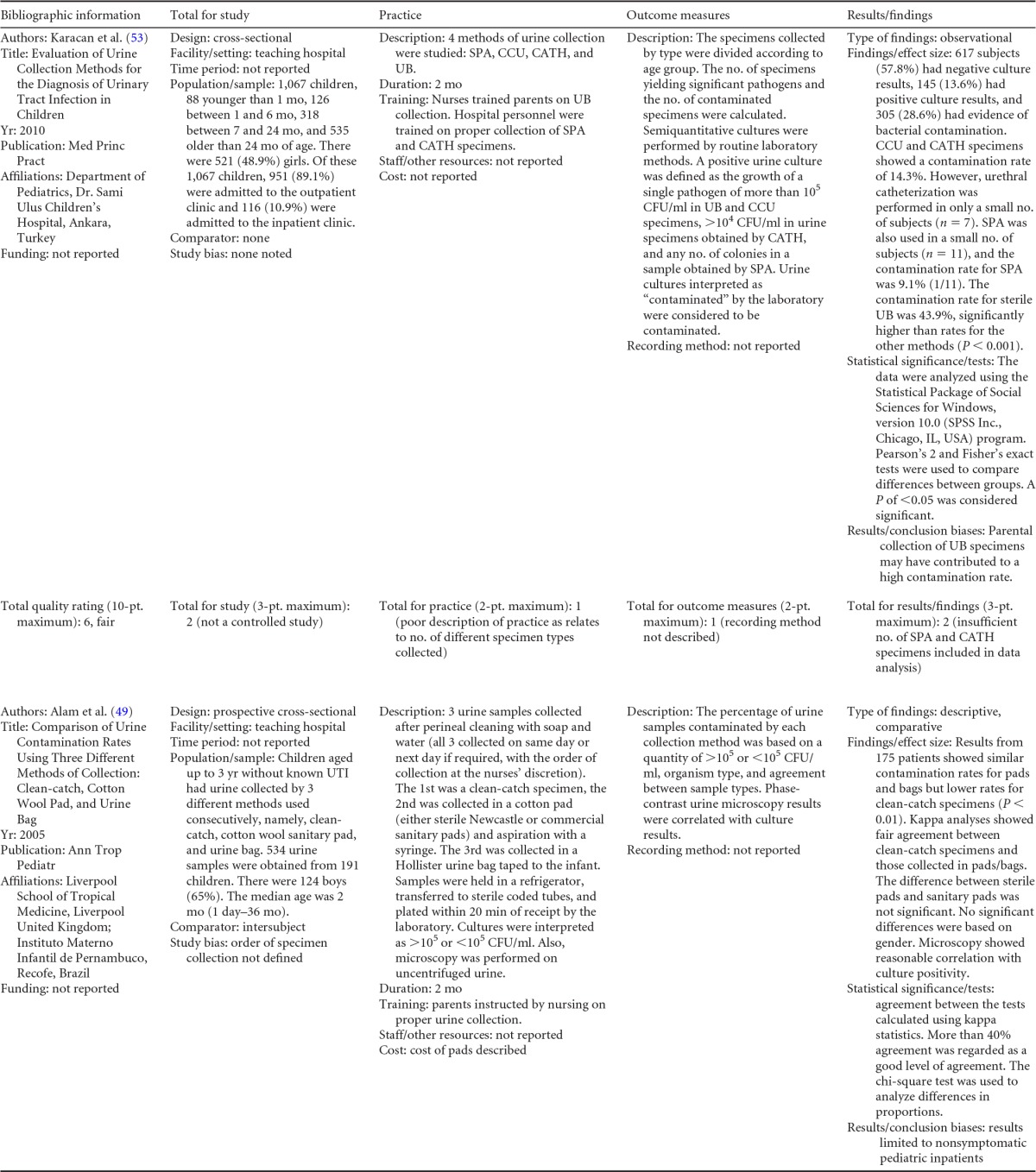

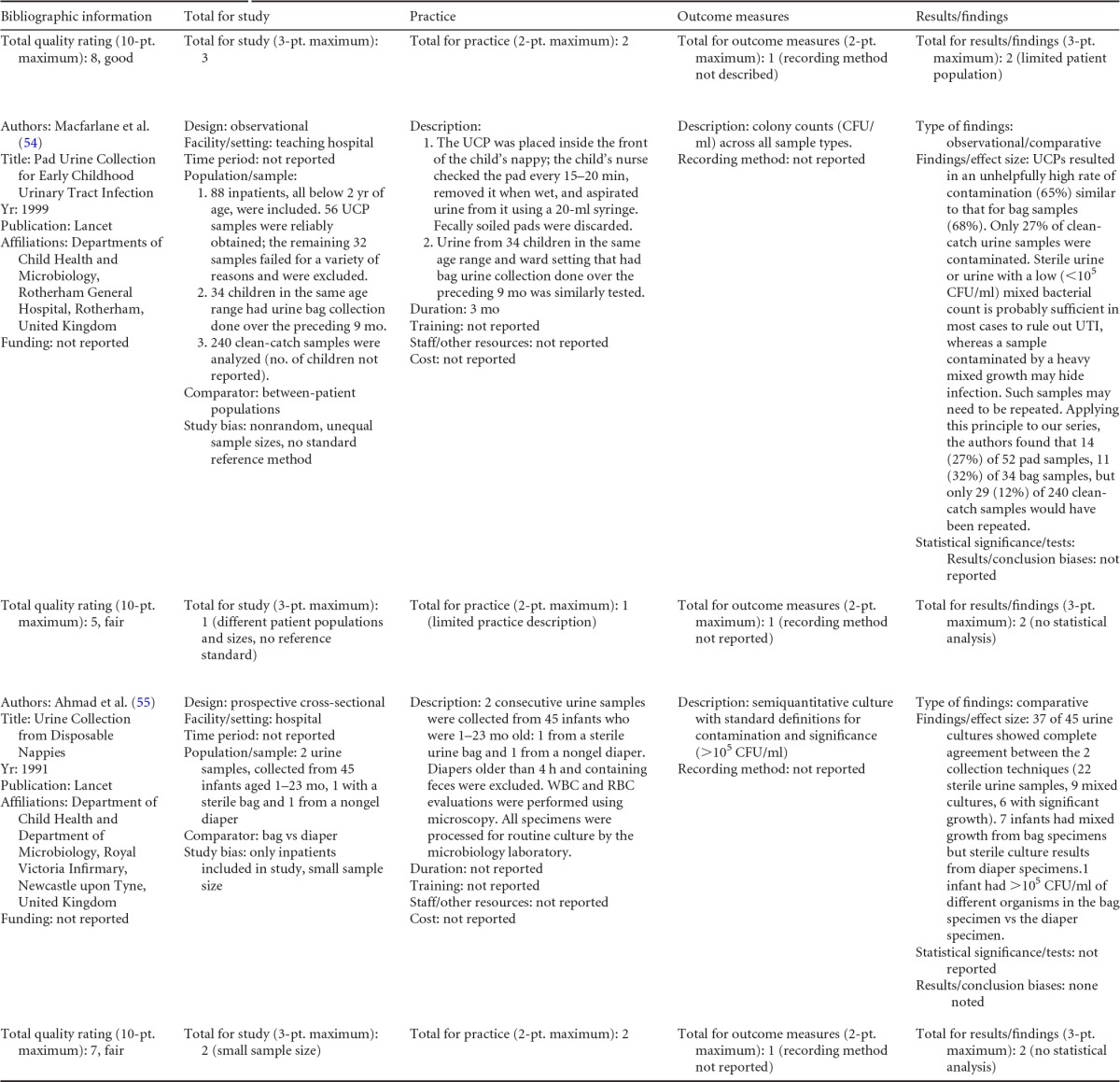

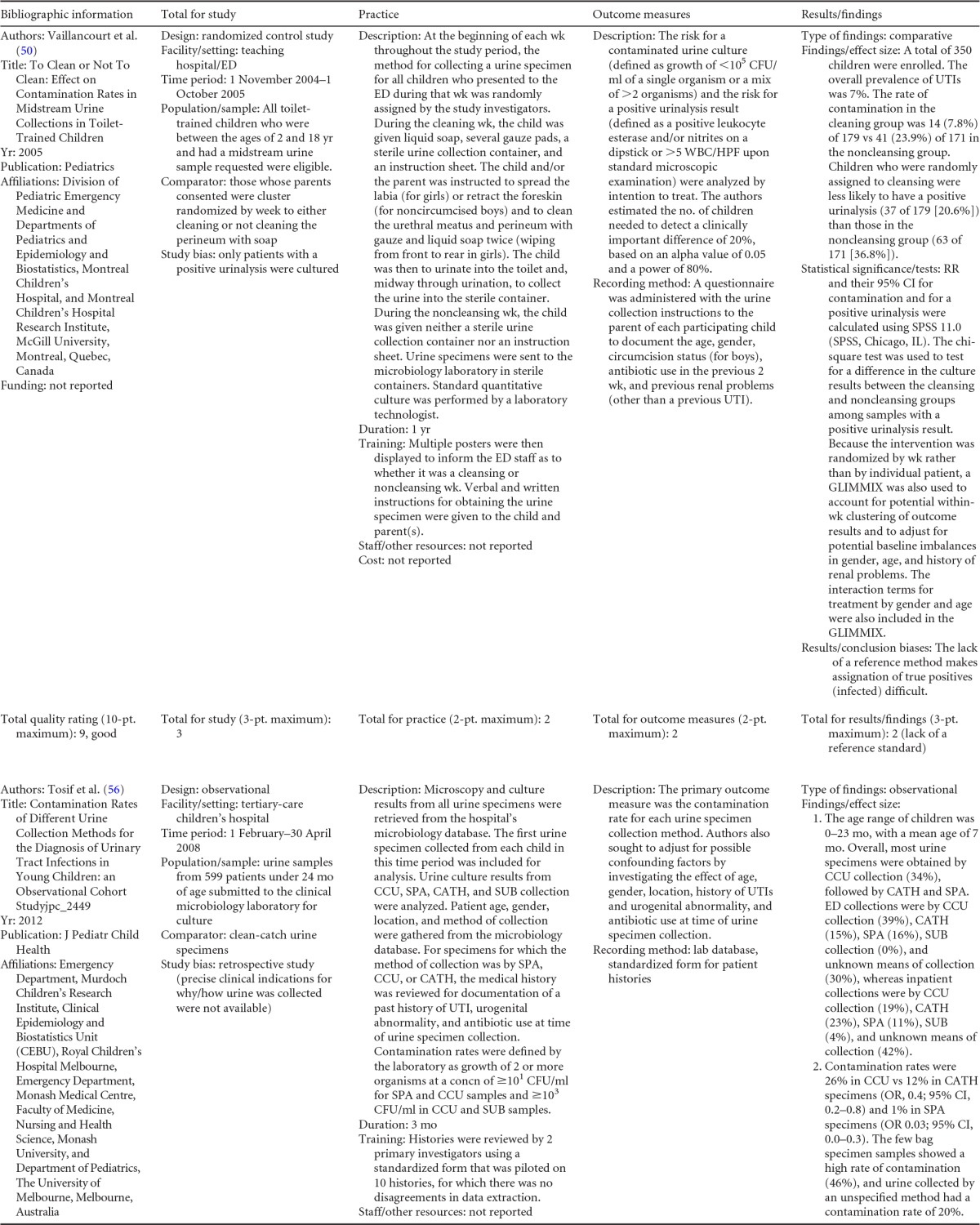

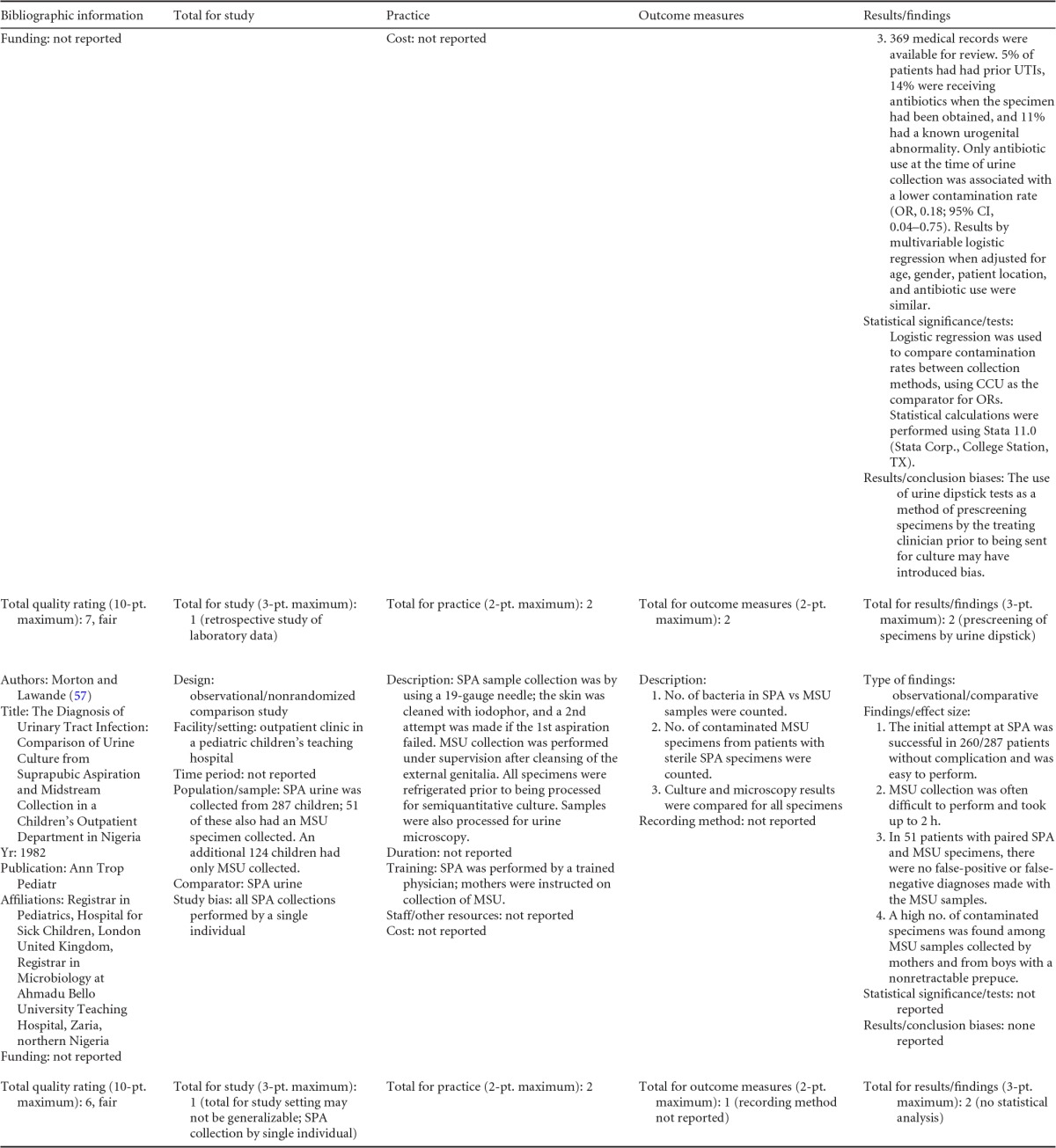

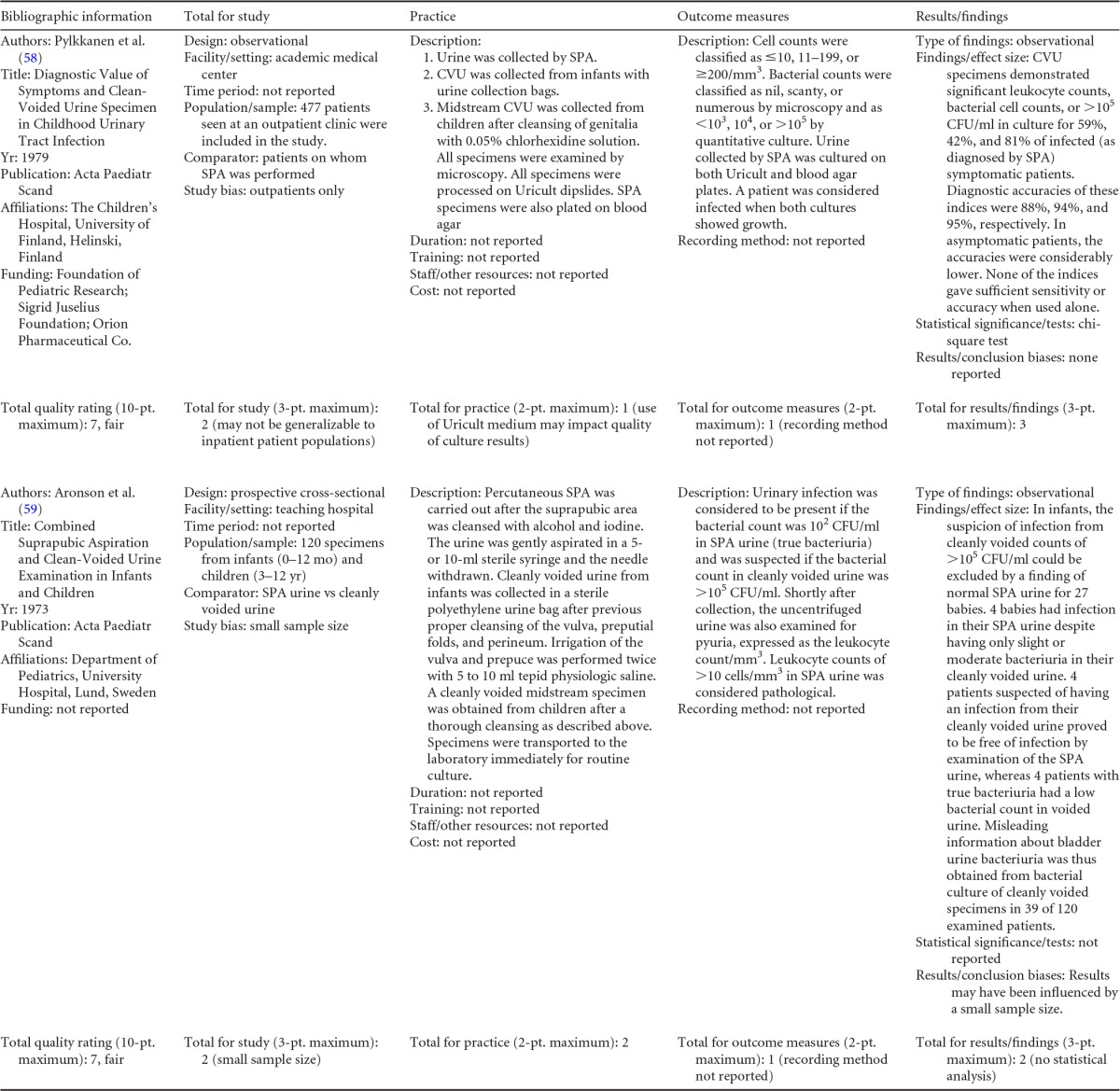

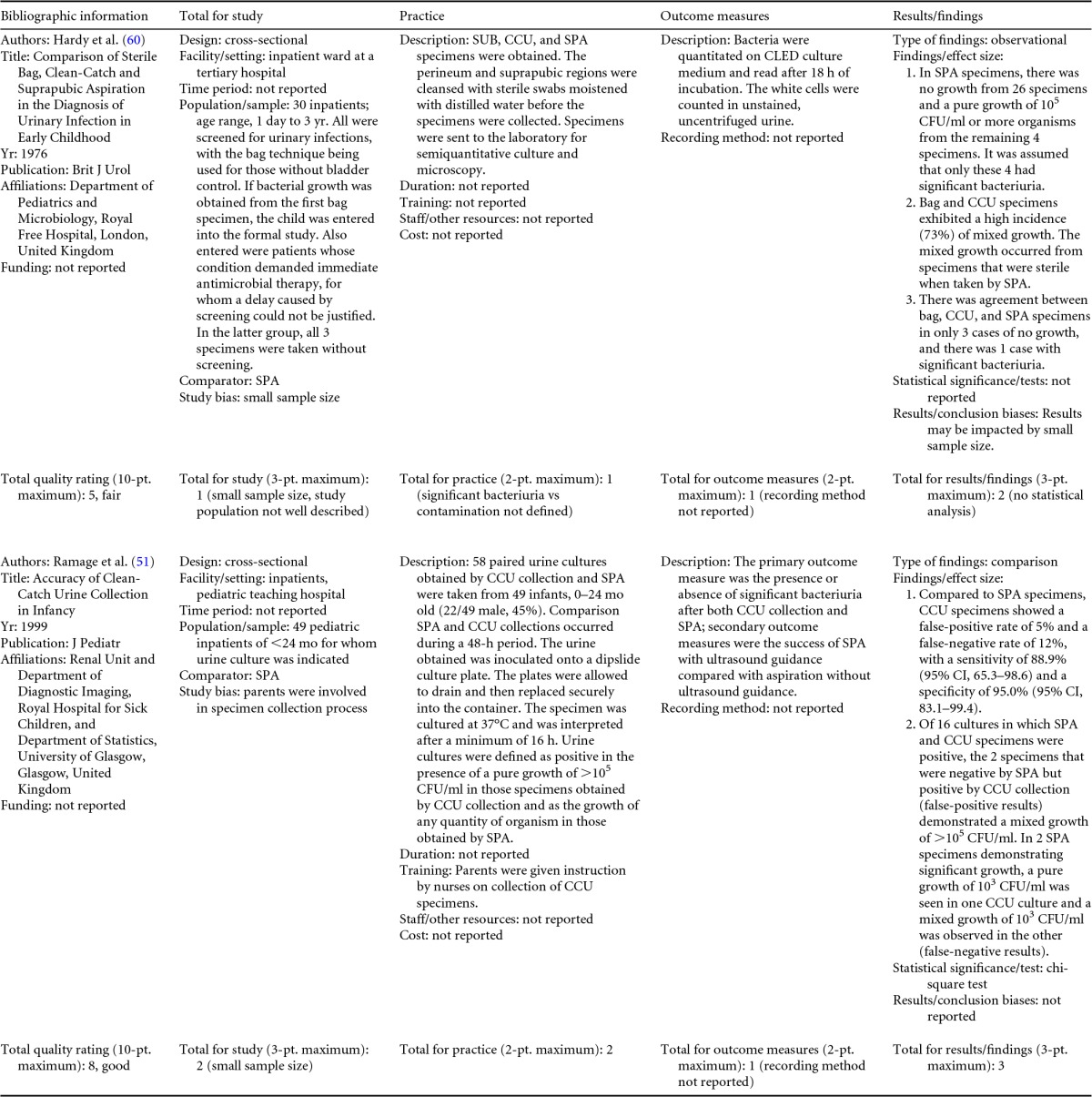

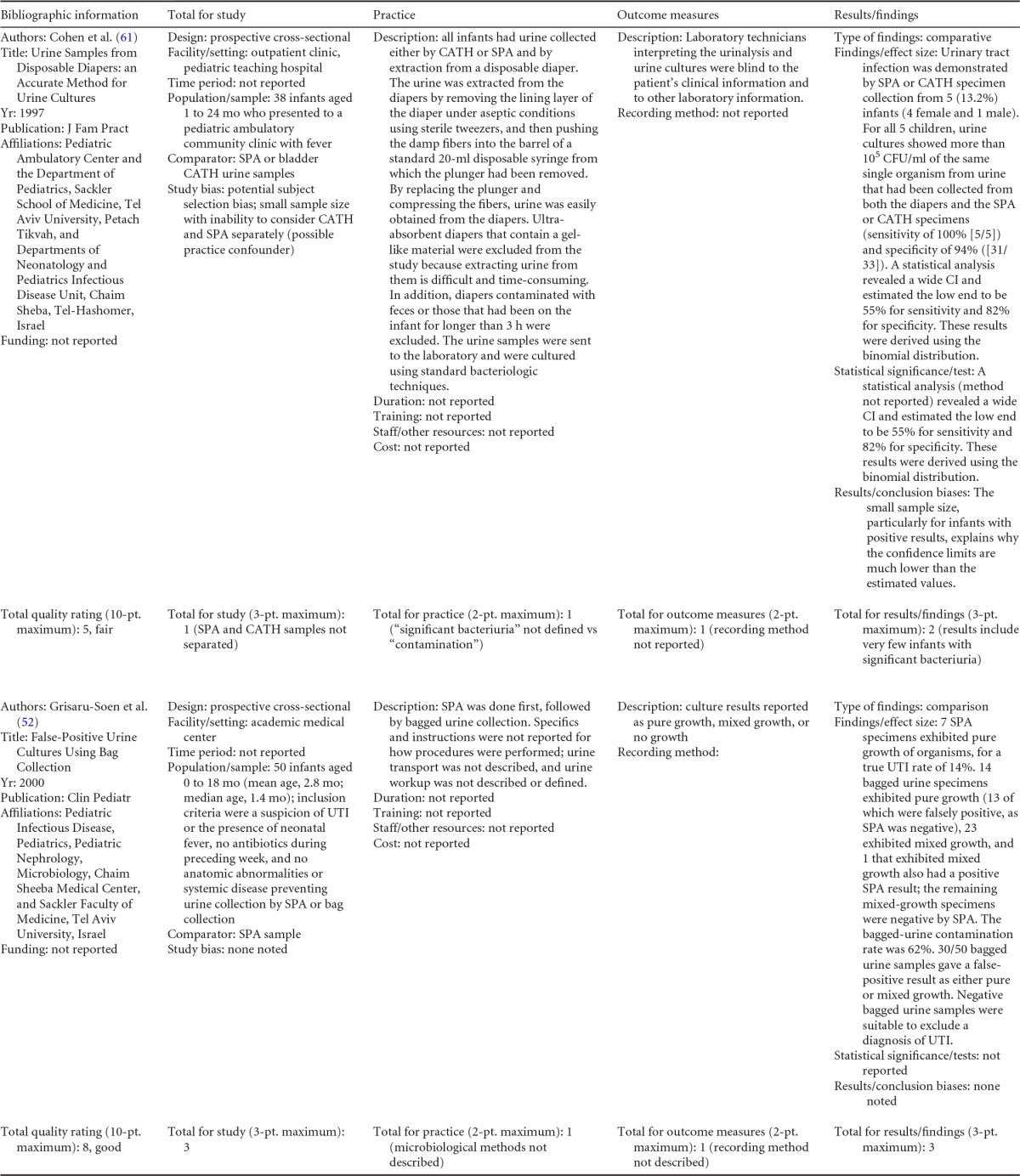

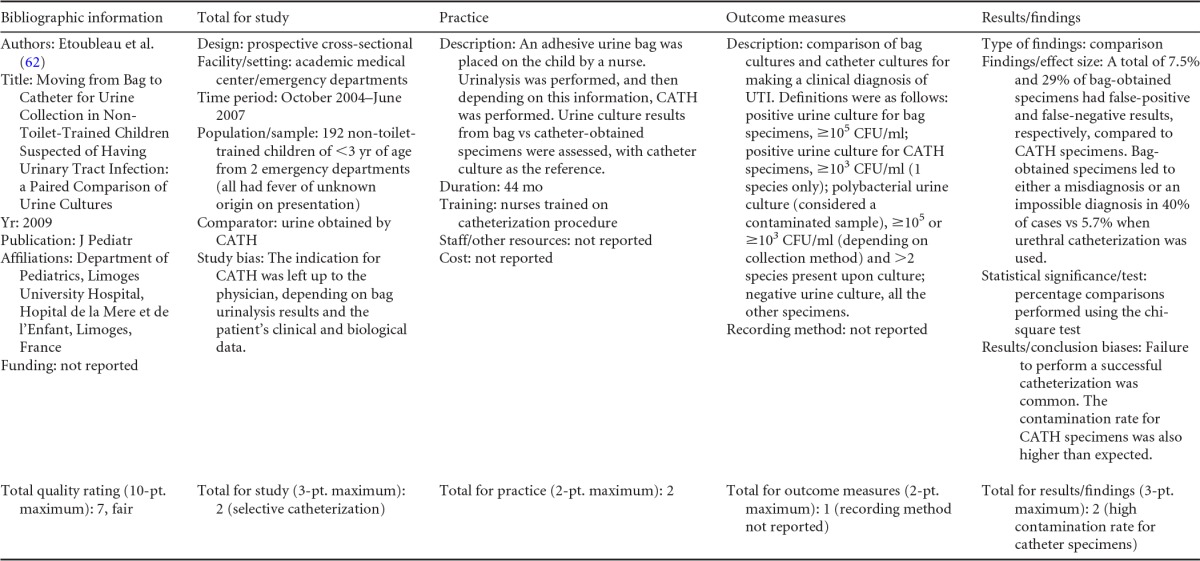
Evidence summary table for contamination and diagnostic accuracy of urine collected from children^*a*^

a SPA, suprapubic aspiration; CCU, clean-catch urine; CVU, cleanly voided urine; CATH, straight catheterization; SUB, sterile urine bag; UCP, urine collection pad; MSU, midstream urine; ED, emergency department; HPF, high-power field; RR, relative risks; 95% CI, 95% confidence interval; GLIMMEX, generalized linear mixed model; OR, odds ratio; CLED, cystine lactose electrolyte-deficient.
